# Nuclear FGF2 orchestrates phase separation-mediated rDNA chromatin architecture to control BMSCs cell fate

**DOI:** 10.1038/s41413-025-00451-y

**Published:** 2025-09-24

**Authors:** Hengguo Zhang, Zifei Wang, Zhenqing Liu, Xuan Li, Wansu Sun, Wenyu Zhen, Fei Xu, Rui Wang, Qi Yin, Shuqin Cao, Mingyue Wu, Jiacai He, Jianguang Xu, Yang Li, Quan Yuan

**Affiliations:** 1 College & Hospital of Stomatology, Anhui Medical University, Key Lab. of Oral Diseases Research of Anhui Province, Hefei, China; 2 State Key Laboratory of Oral Diseases & National Center for Stomatology & National Clinical Research Center for Oral Diseases, West China Hospital of Stomatology, Sichuan University, Chengdu, China; 3 Division of Oral Biology and Medicine, School of Dentistry, University of California, Los Angeles (UCLA), Los Angeles, CA USA; 4 Kidney Disease Center, The First Affiliated Hospital, School of Medicine, Zhejiang University, Hangzhou, China; 5 Department of Stomatology, The First Affiliated Hospital of Anhui Medical University, Hefei, China; 6 Department of Genetics, School of Life Sciences, Anhui Medical University, Hefei, China

**Keywords:** Bone, Bone quality and biomechanics

## Abstract

Ribosomal RNA (rRNA) synthesis is intricately tied to cellular growth and proliferation. Basic fibroblast growth factor (FGF2), a pivotal factor for bone marrow mesenchymal stem cells (BMSCs), can stimulates rRNA transcription, though the underlying mechanism remains unknown. Here, we demonstrate that the cytoplasm-nucleus translocation of FGF2 is determined by the stable nuclear localization motif. Meanwhile, the nuclear FGF2 regulates rRNA expression and BMSCs proliferation via phase separation. Next, through FGF2 related epigenomics and 3D genomes analysis, we identified chromatin architectures during BMSCs differentiation and aging. In the process, topologically associating domains (TADs) and chromatin loops profiling revealed the attenuated genomic interaction among proximal chromosomes 13, 14, 15, 21, and 22, where phase-separated FGF2 facilitates rDNA transcription depend on specific super-enhancers (SEs). Furthermore, we validated that FGF2 orchestrates rDNA chromatin architecture in coordination with STAT5. Together, these findings underscore the pivotal role of FGF2 in rDNA chromatin architectures, which determines BMSCs cell fate.

## Introduction

Inadequate ribosome biogenesis profoundly influences the senescence and altered lineage differentiation potential of bone marrow mesenchymal stem cells (BMSCs), which contribute to age-related disease.^1,2^ Meanwhile, BMSCs exhibit a marked decline in proliferation and encounter disruptions during cell cycle progression,^3,4^ predominantly accompanying with decreased capacity of differentiation and self-renewal.^5,6^ This deviation precipitates a vital linking between ribosome biogenesis and BMSCs cell fate, severely hampering tissue regeneration and repair.^2,7–9^ Consequently, a profound exploration of ribosomal genome stability and dynamics related regulatory mechanisms underpinning BMSCs cell fate is imperative.

As an important component maintaining BMSCs pluripotency and self-renew, basic fibroblast growth factor (FGF2) is initially identified as a mitogen with prominent angiogenic properties.^10,11^ In which, 18-kD isoform FGF2 is deemed to be a critical determinant of bone health,^12^ and *FGF2*^*–/–*^ mice results in significant decreased bone formation.^13,14^ These findings suggest that FGF2 involves a potentially complex molecule or gene regulatory network in bone marrow microenvironment. Importantly, nuclear FGF2 could stimulated ribosomal RNA (rRNA) transcription undergoing cell cycle G0 to G1 transition.^15^ As ribosomal DNA (rDNA) consists of highly repetitive and conserved genomic sequences, which mainly located in the proximal centromere region of chromosomes 13, 14, 15, 21, and 22.^16^ Therefore, rRNA related gene regulation implies efficient and rapid genomic interactions. Despite previous evidences validated that FGF binding FGF receptor activated cell proliferation and differentiation related RAS-RAF-MEK-MAPK pathway,^17^ exploring nuclear located FGF2 to regulate conserved rDNA related chromosome interaction obtain far-reaching significance.

Emerging evidence has linked higher-order chromatin structure to cell-type-specific regulation of cell homeostasis, lineage identity, and cell fate.^18,19^ The eukaryotic three-dimensional (3D) genome is organized in a hierarchical order mainly comprising compartments, topological-associated domains (TADs), and chromatin loops of all shapes and sizes.^20–23^ In the nucleus, different gene regulatory elements are spatially coordinated to regulate complex gene expression^24^. Recent studies indicate that organization of chromatin structure contributes to cell differentiation, including senescence and the altered lineage differentiation potential of stem cells.^25–27^ Meanwhile, a global reduction in chromatin accessibility and plasticity during stem cells lineage commitment and senescence.^28,29^ Structural DNA regulatory elements implicated in cell fate transformation are situated within intergenic or intragenic regions. These elements modulate gene expression by circumventing the linear genome to directly interact with target genes. In this process, phase separation of the master transcription factors or regulatory element have been demonstrated to orchestrate the clustering of chromatin loops, engendering specialized regulatory domains that compartmentalize and concentrate the transcriptional machinery, thereby potentiating high-speed gene expression.^30,31^

Here, through modeling BMSCs differentiation trajectories, we identified that FGF2 play an important role in cell fate transition. Specifical nuclear localization sequences (NLS) determined the nucleolus translocation of FGF2, which further regulated rRNA expression and cell cycle hemostasis by phase separation. Next, we profiled FGF2 related chromatin interactions during BMSCs cell fate transition. In which, we observed FGF2 highly participate in coordinated alterations of rDNA related conservative chromatin TADs and Loops landscape in proximal chromosomes 13, 14, 15, 21, and 22. Mechanistically, nuclear FGF2/STAT5 complex identified specific motifs, which driving the concatenate looping of rDNA and promoting the efficient and rapid expression of rRNA. With remarkable specificity, this study underscores the pivotal role of FGF2 in rDNA related chromatin architecture and rRNA expression, which directing BMSCs cell fate.

## Results

### FGF2 and its nuclear translocation mediates BMSCs cell fate

**Fig. 1:**
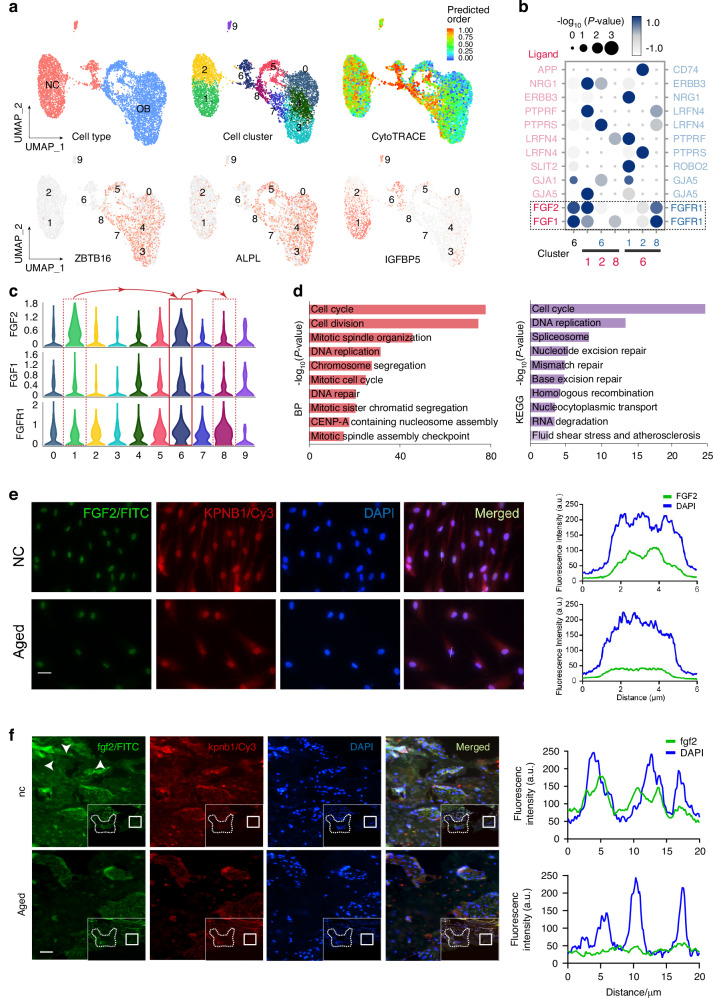
FGF2 and its nuclear translocation mediates BMSCs cell fate. **a** Uniform manifold approximation and projection (UMAP) visualizations of NC (normal control) and OB (osteoblast differentiation) cell clusters, right panel shown the predicted differentiation score, which was calculated by CytoTRACE. **b** CellPhoneDB4 analysis between cell clusters 1, 2, 8, and cell cluster 6, focusing on their roles as ligands and receptors, respectively. **c** Violin plots further showing the expression distribution of FGF2, FGF1, and FGFR1 across different cell clusters. **d** Gene Ontology (GO) biological process (BP) and KEGG enrichment analysis for differentially expressed genes in cluster 6. The bar plots display the top 10 enriched terms. **e** Colocalization of FGF2 and KPNB1 was detected by immunofluorescence in normal and aged human BMSCs. Scale bar, 20 μm. **f** Colocalization of fgf2 and kpnb1 was detected by immunofluorescence in normal and aged mouse alveolar bone. Scale bar, 40 μm

To determine the pivotal molecules and underlying mechanisms during BMSCs cell fate transition, we integrated multi-omics experiments and analyses containing single-cell transcriptome (GSE113253), proteome, epigenome, 3D genome in normal BMSCs, terminal differentiation BMSCs, and aging BMSC. In which, we profiled the cellular and molecular trajectories during osteogenic differentiation (including undifferentiation and osteogenic induction for 7 days), which represents the archetypal terminal differentiation. Dimensional reduction analysis using uniform manifold approximation and projection (UMAP) revealed a diversity of normal and osteogenic BMSCs cell types and related differentiation trajectories (Fig. 1a, Fig. S1A).^32^ The osteogenic induction BMSCs consistently exhibited robust expression of ZBTB16 and ALPL, while the undifferentiated cell cluster 6 is the group of osteoprogenitor cells at the starting point of differentiation (Fig. 1a).

FGF family, especially FGF2 serves as a crucial bridging role in facilitating intercellular communication and specifically high expressed during osteogenic differentiation (Fig. 1b, c). Meanwhile, the pro-osteoblast cell cluster 6 demonstrated dynamic cell cycle activity and mitotic spindle assembly (Fig. 1d). Importantly, consist with osteogenic differentiation, the expression of FGF2 (mainly 18-kD molecular weight) decreased significantly in aged BMSCs and regenerated alveolar bone from aged mouse (Fig. S1B). Furthermore, FGF2 is mainly specific distributed and concentrated in the nuclei of normal BMSCs (Fig. 1e) and alveolar bone (Fig. 1f). Meanwhile, FGF2 nuclear import from the cytosol requires the classical nuclear import machinery, involving proteins karyopherin.^33^ As expected, we discover the colocalization of FGF2 and karyopherin beta 1 (KPNB1) in the nucleus (Fig. 1e, f). After KPNB1 knockdown, protein level of 18-kD FGF2 was not notably changed, while the nuclear translocation was markedly reduced (Fig. S1C, S1D). These collective results indicate that the nuclear translocation of 18-kD FGF2 facilitates intercellular communication and functions as a potentially pivotal mediator during the fate transformation of BMSCs.

Given that FGF2 is a secreted protein, its nuclear localization is usually mediated by short sequences of basic amino acids known as NLS and forms stable complexes with nuclear pore transport proteins, ultimately being translocated into the nucleus to exert its functions.^34^ Docking of the importin or substrate complex to the nuclear pore complex (NPC) is mediated by karyopherin, which can bind to nuclear localization sequences in cargo substrates or act as an autonomous nuclear transport receptor.^35,36^ In view of this, it is important to explore the NLS motif characteristics of FGF2, which contribute to effectively supplementing FGF2 and maintaining BMSCs cell cycle and lineage commitment.

### Nuclear FGF2 regulates BMSCs rRNA synthesis

**Fig. 2:**
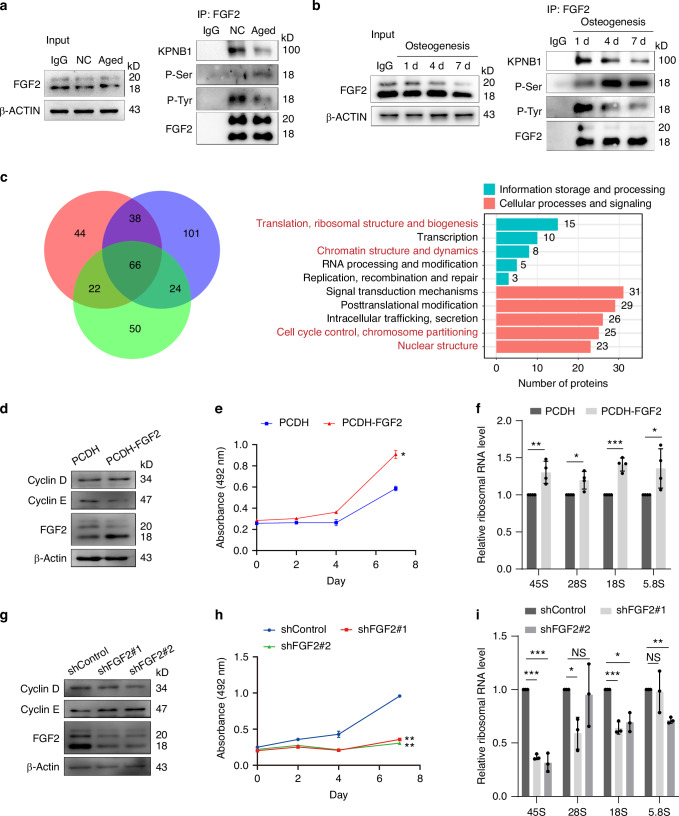
Nuclear FGF2 regulates BMSCs rRNA synthesis. **a** Immunoprecipitation of KPNB1 and p-Tyr/Ser using an anti-FGF2 antibody in normal and aged BMSCs. **b** Immunoprecipitation of KPNB1 and p-Tyr/Ser using an anti-FGF2 antibody in BMSCs undergoing osteogenic induction at 1 day, 4 days, and 7 days. **c** Gene Ontology (GO) functional annotation analysis for biological processes occupied by FGF2. **d** Western blotting against CCND1, CCNE1, and FGF2 in BMSCs after transduction with PCDH and PCDH-FGF2. **e** CCK8 assays were performed in BMSCs after transduction with PCDH and PCDH-FGF2. **f** RT-qPCR analysis of 45S rRNA, 28S rRNA, 18S rRNA, and 5.8S rRNA levels in BMSCs after transduction with PCDH and PCDH-FGF2. **g** Western blotting against CCND1, CCNE1, and FGF2 in BMSCs after transduction with shControl, shFGF2#1, or shFGF2#2. **h** CCK8 assays were performed in BMSCs after transduction with shControl, shFGF2#1, or shFGF2#2. **i** RT-qPCR analysis of 45S rRNA, 28S rRNA, 18S rRNA, and 5.8S rRNA levels in BMSCs after transduction with shControl, shFGF2#1, or shFGF2#2. *P < 0.05; **P < 0.01; ***P < 0.001; NS *P* > 0.05 by unpaired two-tailed Student’s *t* test

Phosphorylation of NLS can significantly modulate the nuclear import, while tyrosine phosphorylation is critical for maintaining the stability of protein complexes.^37–39^ FGF2 engage specifically with tyrosine kinase receptors to initiate FGFR signaling.^40–42^ To explored the nuclear transport mechanism of FGF2, we conducted immunoprecipitation assays during BMSCs aging and osteogenic differentiation. As the low expression levels of FGF2 in aged or terminal differentiation BMSCs, the interaction between FGF2 and KPNB1 was significant declined (Fig. 2a, b). Meanwhile, tyrosine phosphorylation levels of FGF2 were decreased, in contrast to marked increase of serine phosphorylation levels (Fig. 2a, b). By protein electrophoresis and 18-kD position related mass spectrometry (MS), we further demonstrated that phosphorylation of FGF2 Serine 255 was specific abounded in aged BMSCs (Fig. S2A, S2B). Moreover, the NLS Mapper (http://nls-mapper.iab.keio.ac.jp/) and NetworKIN (http://networkin.info/) indicated that the Ser 255 was in NLS, which was essential for the nuclear translocation of FGF2 (Fig. S2C).

To investigate the function of nuclear FGF2, we implemented immunoprecipitation assays and MS in nuclear lysates of BMSCs (Fig. S2D). In which, Gene Ontology (GO) enrichment analysis demonstrates that nuclear FGF2 is predominantly implicated in ribosomes biogenesis, transcription, chromatin structure & dynamics, signal transduction mechanisms, cell cycle, cell growth, etc. (Fig. 2c) Furthermore, overexpression of FGF2 in BMSCs was associated with enhanced cell cycle activity, cell viability, and ribosomal RNA expression (Fig. 2d–f). Conversely, knockdown of FGF2 led to marked cell cycle arrest and diminished cellular proliferation viability, concomitant with a decrease in ribosomal RNA expression (Fig. 2G–I). Taken together, these findings demonstrated that nuclear transport FGF2 regulated the cell cycle and ribosomal RNA expression during BMSCs cell fate transition.

### FGF2 regulates rRNA synthesis via phase separation

Given the pivotal role of FGF2 in modulating ribosomal RNA expression in BMSCs, we further explored the mechanisms whereby FGF2 facilitates rDNA transcription. As FGF2 appears in a markedly condensed form in the nucleus of BMSCs (Fig. 1d) and phosphorylation commonly influence the polymerized state of protein, we hypothesized that the FGF2 condensates enriched at specific nucleolus regions via phase separation and contribute to rDNA transcription. Based on PhaSepDB^43^ (http://predict.phasep.pro/) prediction for the domain and feature of FGF2 protein sequence, we found that FGF2 exhibits significant phase separation capability, and NLS motif region enriched with prion-like structure and phosphorylation site (Figs. S3A, 3B, Fig. S2C).

**Fig. 3:**
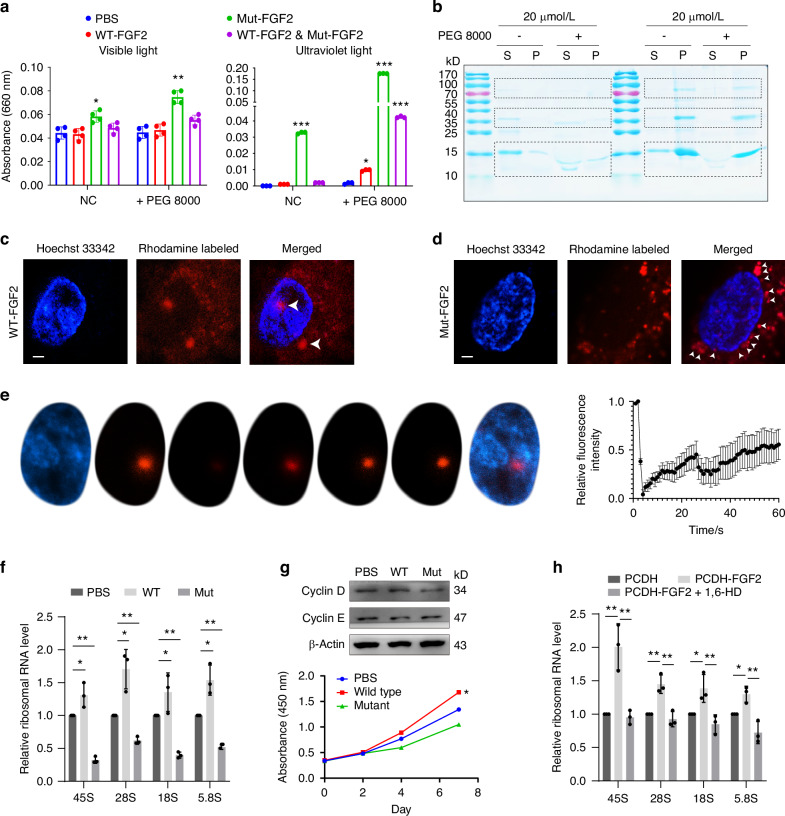
FGF2 regulates rRNA synthesis via phase separation. **a** Determination of turbidity of wild-type-FGF2 and mutant-FGF2 in vitro in the presence or absence of 20% PEG 8000 with microplate reader (left panel) and ultraviolet detector (right panel). All experimental groups were compared with PBS group. **b** Sedimentation assay of FGF2. Representative Coomassie brilliant blue staining of wild-type-FGF2 and mutant-FGF2 fractions after centrifugation in the presence or absence of 20% PEG 8000. S: supernatant, P: re-suspended pellet. Unimer (~18 kD) and estimated Dimer (~35 kD) and Tetramer FGF2 ( ~ 70 kD) can be observed. **c**, **d** BMSCs incubating with 50 ng/mL Rhodamine B labeled FGF2 for 24 h, left panel: wild-type FGF2, right panel: mutant FGF2, Scale bar, 2 μm. **e** FRAP assay after incubating BMSCs with 50 ng/mL Rhodamine B labeled wild-type FGF2 for 24 h. Quantified FRAP rate of FGF2 condensate, right panel. Data represents the mean ± SD of 3 independent condensates. **f** RT-qPCR analysis of 45S rRNA, 28S rRNA, 18S rRNA, and 5.8S rRNA levels in BMSCs treated for 24 h with PBS, wild-type-FGF2 (50 ng/mL), and mutant FGF2 (50 ng/mL). **g** Top panel shows western blotting against proliferation-associated cyclins in BMSCs treated for 24 h with PBS, wild-type-FGF2 (50 ng/mL), and mutant FGF2 (50 ng/mL). bottom panel shows CCK8 assays in BMSCs treated for 24 h with PBS, wild-type-FGF2 (50 ng/mL), and mutant FGF2 (50 ng/mL). **h** RT-qPCR analysis of 45S rRNA, 28S rRNA, 18S rRNA, and 5.8S rRNA levels in BMSCs after transduction with PCDH and PCDH-FGF2 in combination with 1,6-hexanediol treatment. *P < 0.05; **P < 0.01; ***P < 0.001 by unpaired two-tailed Student’s *t* test

To reinforce FGF2 condensation in a cell-free system, recombinant wild-type FGF2 and Ser 255 mutation FGF2 were constructed (Fig. S3C). We found that both wild-type FGF2 and mutant FGF2 forms distinct dimers and even tetramers under non-denaturing conditions, compared to the condition of denaturation at 95 °C (Fig. S3D). Meanwhile, the aggregation of FGF2 was more pronounced in mutant FGF2 (Fig. S3D). By measurement the turbidity based on visible light and ultraviolet light, the turbidity of wild-type FGF2 markedly increased in a PEG concentration-dependent manner (Fig. 3a). Furthermore, sedimentation assays validated that wild-type FGF2 was present in the precipitate after PEG addition, whereas the mutant FGF2 always resided in the precipitate, suggesting that mutant FGF2 has the propensity to form protein polymerization (Fig. 3b). AlphaFold 2 related modeling further revealed mutant FGF2 forms substantial molecular hydrogen bonds, which promotes the propensity for protein aggregation (Fig. S3E). Moreover, we have further validated the phase separation capacity of FGF2 by performing in vitro droplet formation assays. Following rhodamine conjugation and subsequent dialysis, FGF2 variants exhibited distinct biophysical behaviors: mutant FGF2 displayed irreversible protein aggregation, while wild-type FGF2 maintained stable dispersion (Fig. S3F). Under 300 mmol/L NaCl with PEG8000, WT-FGF2 formed distinct liquid-like droplets in vitro, indicative of phase separation. In contrast, the mutant FGF2 failed to form spherical droplets and instead exhibited irregular aggregates under the same conditions (Fig. S3G). This observation aligns with our AlphaFold2-based structural modeling (Fig. S3E), which revealed that the mutant FGF2 forms excessive intramolecular hydrogen bonds.

By labeling the recombinant wild-type FGF2 protein with rhodamine, we observed distinct phase-separated condensates in BMSCs, particularly within the nucleus (Fig. 3c). In contrast, the mutated FGF2 predominantly accumulates around the periphery of the nucleus, with limited entry into the nuclear compartment (Fig. 3d). Fluorescence recovery after photobleaching (FRAP) analysis exhibited that wild-type FGF2 form droplets in the BMSCs nucleus and rapidly recovered from photobleaching (Fig. 3e).

To investigate the potential rDNA transcription-responsive functions of FGF2, we detected rRNA expression and cell cycle in exogenous FGF2 addition and endogenous FGF2 with phase separation treatment, respectively (Fig. 3f–h). We observed that the presence of wild-type FGF2 significantly facilitates the transcription of rDNA and enhances cell cycle activity and cell viability, whereas the mutant type of FGF2 exerts inhibitory effects (Fig. 3f, g). The addition of 1,6-hexanediol completely abrogated the enhancement in rDNA transcription activity induced by FGF2 overexpression (Fig. 3h). These results demonstrate that phase separation-driven FGF2 condensation plays a pivotal role in regulating rDNA transcription in BMSCs.

### FGF2 involves in rDNA regions related epigenetic regulation

**Fig. 4:**
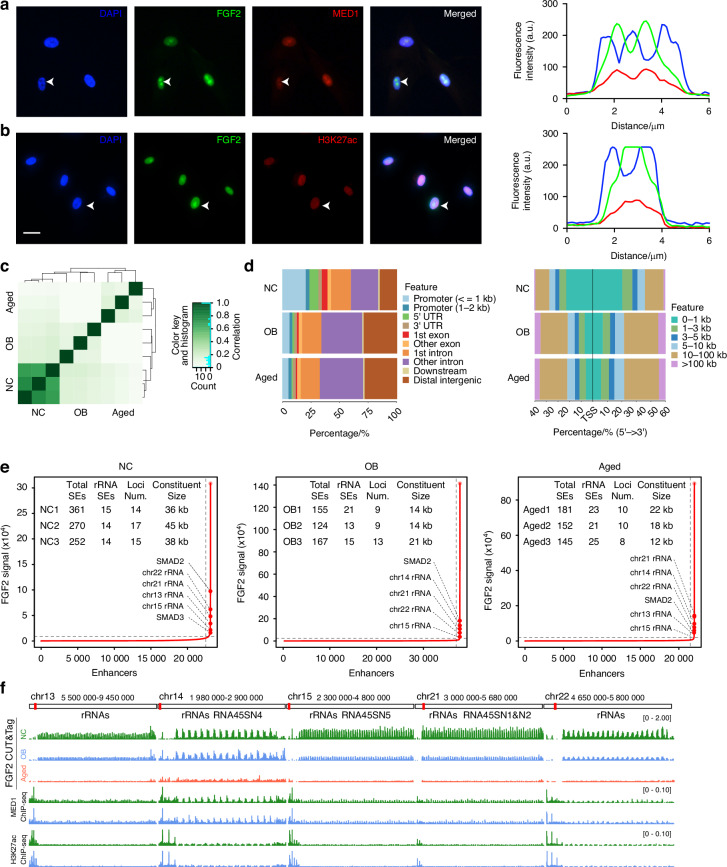
FGF2 involves in rDNA regions related epigenetic regulation. **a**, **b** Colocalization of FGF2 and MED1 or H3K27ac was detected by immunofluorescence in normal BMSCs. Scale bar, 10 μm. **c** Correlation matrix of FGF2 CUT&TAG data across three groups: NC, OB, and aged. The color gradient represents correlation coefficients, with dark green indicating high correlation (close to 1) and light green indicating lower correlation (close to 0). **d** Stacked bar charts representing the genomic distribution of FGF2 binding sites (left panel) and their distance to transcription start sites (TSS) (right panel) in NC, OB, and aged groups. **e** Hockey stick plots illustrate the rank order of FGF2 signals across all enhancers in three conditions: normal BMSCs, differentiated BMSC and aged BMSCs. Inserted panels display the number of total super-enhancers (SEs) and ribosomal RNA super-enhancers (rRNA SEs), as well as the constituent size of SEs in three sets of biological replication data. **f** Integrative Genomics Viewer (IGV) visualization of FGF2-seq read density in rRNA regions across normal, differentiated, and aged BMSCs, presented in three sets of biological replicates

Transcriptional coactivators (such as BRD4 and MED1) undergoing phase separation to engender droplets at super-enhancers (SEs) regions, thus facilitating the congregation of transcriptional machinery and effectuating compartmentalization of the transcriptional process.^30^ As nuclear transported FGF2 promote rDNA transcription through phase separation, we further observed that FGF2 exhibits pronounced colocalization with MED1 and H3K27Ac (Fig. 4a, b). To investigate the potential epigenetic regulation of FGF2, anti-FGF2 related CUT&TAG generated in nuclear lysate of normal BMSCs, osteogenic BMSCs, and aged BMSCs (Figs. 4c, S4A). Compared to differentiated and aged BMSCs, normal BMSCs enriched with large proportion of FGF2 binding sites in promoter (≤1 kb), 5’ UTR, and Exon regions, while these sites are predominantly located closer to the transcription start site (TSS) (0–1 kb) (Fig. 4d). Conversely, the differentiated and aged BMSCs obtained abundant FGF2 binding sites in intron and distal intergenic regions and distributed at long distances from the TSS (Fig. 4d). Meanwhile, the number of FGF2-dependent differentially expressed genes (DEGs) significantly increased in normal BMSCs (Fig. S4B), which exhibited more activity peaks near the TSS and represented higher transcriptional activity (Fig. S4C). Moreover, the Kyoto Encyclopedia of Genes and Genomes (KEGG) analysis highlights a substantial number of pathways related to ribosome, nucleocytoplasmic transport, ribosome biogenesis, cell cycle, and cellular senescence (Fig. S4D). Additionally, Gene Ontology (GO) analysis showed abundant pathway related to protein binding, nucleotide binding, ribonucleotide binding, ribonucleoside binding, and nucleoside binding (Fig. S4D).

Super-enhancers (SEs) commonly characterized by extraordinarily long distances and high concentrations of transcriptional machinery, specifically involved in cell development, and differentiation.^44–47^ By using the ROSE (Rank Ordering Of Super-enhancers) algorithm, rDNA regions related chromosomes 13, 14, 15, 21, and 22 were significantly enriched super-enhancers, along with the transcriptional activator SMAD2 (Fig. 4e). Additionally, the data indicate that the number of super-enhancers associated with rRNA genes tends to increase during aging process, while the loci number and constituent size decreases in aged and differentiated BMSCs (Fig. 4e). Furthermore, the Integrative Genomics Viewer (IGV) revealed epigenetic concordance of FGF2, MED1, and H3K27AC within rDNA regions. Importantly, more pronounced FGF2-related peaks were observed in the normal BMSCs, signifying enhanced rDNA transcriptional activity (Fig. 4f).

### Chromatin architectures feature of rDNA regions during BMSCs cell fate transition

**Fig. 5:**
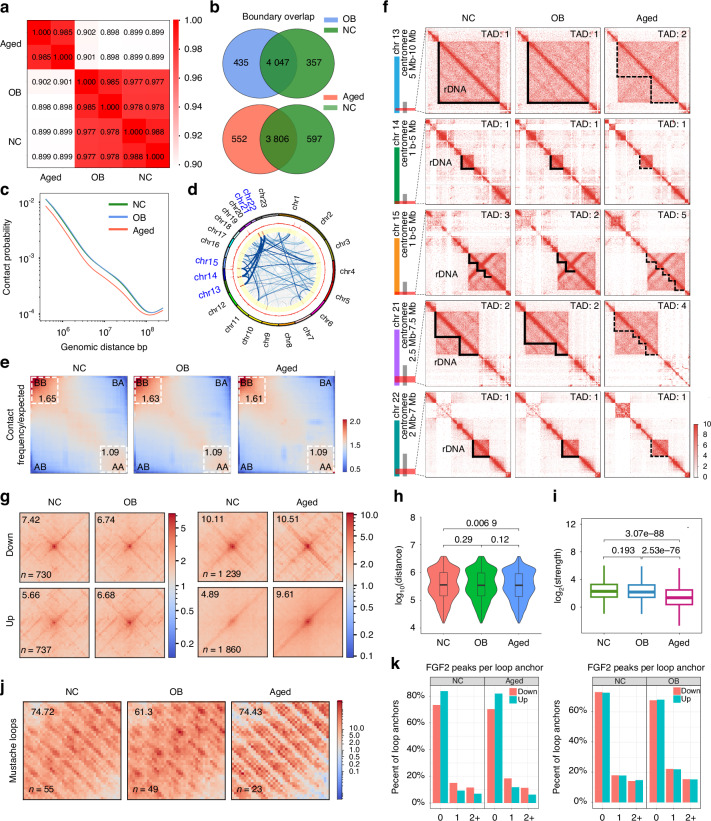
Chromatin architectures feature of rDNA regions during BMSCs cell fate transition. **a** Heatmap of Hi-C interaction matrices for NC, OB, and aged groups. The color gradient represents the correlation coefficients between interaction matrices. **b** Venn diagrams showing the overlap of TAD boundaries between NC and OB BMSCs (top), and between NC and aged BMSCs (bottom). The numbers in each section represent the count of unique and shared boundaries between the compared conditions. **c** Relationship between contact probability observed by Hi-C relative to linear genomic distance in NC, OB and aged groups. **d** Cir-cos diagram showing remarkable inter-chromosomal interactions of chr13, chr14, chr15, chr21 and chr22. **e** Saddle plots showing the compartmentalization of chromatin contacts in NC, OB, and aged BMSCs. The maps depict the observed contact frequency relative to the expected frequency across compartments. The quadrants represent the four possible compartment combinations: AA, AB, BA, and BB. The color gradient from blue to red reflects the range of contact frequency ratios, with red indicating higher-than-expected contact frequencies and blue indicating lower-than-expected frequencies. **f** Hi-C contact maps of rDNA regions across five chromosomes (chr 13, chr 14, chr 15, chr 21, chr 22) in NC, OB, and aged groups. The maps show topologically associating domains (TADs) with rDNA regions highlighted in black. Solid black lines indicate stable TADs across conditions, while dashed black lines represent changes in TAD structure specific to the aged group. **g** Aggregate NC and OB Hi-C signals of downregulated and upregulated chromatin loops. Difference between NC and aged group aggregate signals is shown on the right. **h** Violin plots showing the distribution of log10-transformed chromatin loop distances in NC, OB, and aged groups. The plots display the density of loop distances, with an embedded boxplot indicating the median and interquartile range. **i** Boxplots comparing the log2-transformed interaction strength of chromatin loops in NC, OB, and aged groups. The boxplots display the median, interquartile range, and the overall distribution of loop strengths in each group. **j** Heatmaps showing the enrichment of Mustache-detected loops in NC, OB, and aged groups. **k** Bar charts displaying the percentage of loop anchors with FGF2 peaks in NC, OB, and aged groups. The left panel compares NC with aged BMSCs, and the right panel compares NC with OB BMSCs. The x-axis indicates the number of FGF2 peaks per loop anchor. The y-axis shows the percentage of loop anchors in each category

As the distinct chromatin architecture during BMSCs differentiation and aging, we further assess the three-dimensional (3D) genome of the rDNA regions through high-throughput chromosome conformation capture (Hi-C) (Fig. 5a). Compared with the aging group, the normal and osteoblast differentiation (OB) groups obtained more overlapping boundaries (Fig. 5b). Notably, genome-wide distance-dependent chromatin interaction frequencies showed average contacts decayed more strongly with distance in the aged group compared to the normal or OB groups, as observed in the Hi-C contact matrices across all distances (Fig. 5c). Importantly, above 30% globally inter-chromosomal interactions occurred among proximal chromosomes 13, 14, 15, 21, and 22 in all groups (Fig. 5d).

Next, we considered whether the fate transition of BMSCs might influence the segregation of domains into nuclear compartments, we observed a diminished compartmentalization within the inactive (B) compartments of aged BMSCs, while the active (A) compartments remained largely unaffected (Fig. 5e). Concretely, in aged BMSCs, dynamic modifications in genomic compartments resulted in notable shifts in compartmental affiliation scores, with 5% transitioning from A to B and 6.8% transitioning from B to A (Fig. S5A). Under differentiation conditions, these changes were 4.1% and 3.5%, respectively (Fig. S5B). Additionally, the fate transition of BMSCs showed significant reorganization of topologically associating domains (TADs). The aging process caused 18.6% of TADs in NC group to be either split, merged, or rearranged in aged group. Under osteogenic differentiation conditions, this proportion was approximately 12.8% (Fig. S5C, S5D). Moreover, TADs in NC group that split into smaller TADs in OB and aged group were on average twice the size of invariant TADs (Fig. S5C, S5D), suggesting that they were composed of two underlying TADs connected by undifferentiation property-dependent contacts that spanned a common border. By local focus of TADs on the proximal centromeres, the interaction intensity of the rDNA TAD markedly diminished and discontinuous in the aged group (Fig. 5f).

Through global aggregate peak analysis (APA) and heatmap analysis of Hi-C contact frequencies, we substantiated that differential Hi-C chromatin loops signify substantial alterations in contacts between NC and OB groups, as well as between the NC and aged cohorts (Fig. 5g). Subsequent experiments elucidated that chromatin loops were short-distance and low-strength in the aged group compared to the NC or OB groups (Fig. 5h, i). By local APA analysis of rDNA regions, in contrast to NC group, loop strength decreased in OB group while loop counts significantly reduce in aged group (Figs. 5j, S5E). Additionally, the distribution of FGF2 peaks overlapping the anchors of differential loops revealed a higher consistency between NC and OB group (Fig. 5k). These findings underscore rDNA regions related the three-dimensional chromatin architectures during BMSCs cell fate transition, which are consistent with FGF2 epigenomics characteristics.

### Phase separation FGF2 orchestrates rDNA region related chromatin architecture

**Fig. 6:**
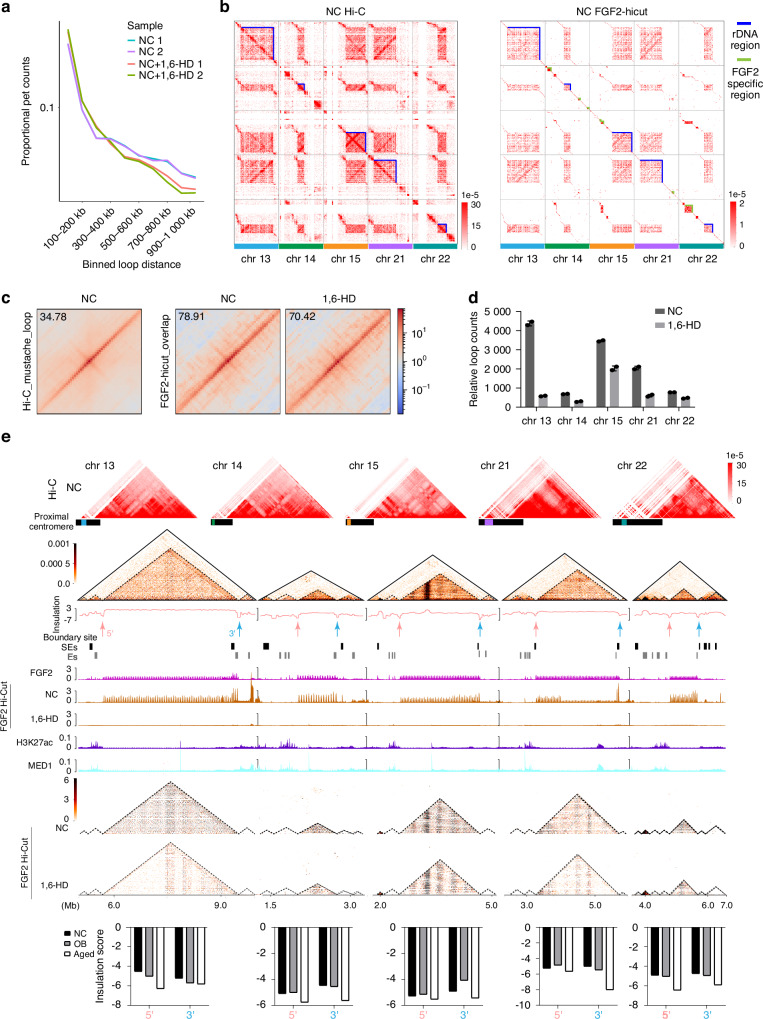
Phase separation FGF2 orchestrates rDNA region related chromatin architecture. **a** Line plots showing the proportional pet counts across different binned loop distances in NC and NC + 1,6-HD treated BMSCs. The x-axis represents the binned loop distances (100–200 kb to 900–1 000 kb), and the y-axis shows the proportional pet counts on a logarithmic scale. Each line represents a biological replicate. **b** Hi-C (left) and FGF2-hicut (right) contact maps for normal BMSCs across five chromosomes (chr 13, chr 14, chr 15, chr 21, chr 22). The Hi-C map (left) displays the overall chromatin interaction frequencies, with blue boxes highlighting the rDNA regions. The FGF2-hicut map (right) shows chromatin interactions specific to FGF2, with green boxes indicating regions of significant FGF2 binding. The color gradient represents the interaction frequencies, with red indicating higher contact frequencies. **c** Aggregate peak analysis (APA) results obtained by all HiC loops on HiC matrix (left) and HiCUT loops overlapping with Hi-C on the Hi-C matrix (middle and right). The color gradient represents interaction frequencies, with red indicating higher frequencies and blue indicating lower frequencies. **d** Bar chart comparing the relative loop counts across five chromosomes (chr 13, chr 14, chr 15, chr 21, chr 22) in NC and 1,6-HD-treated BMSCs. Each bar represents the mean loop count for each chromosome. **e** Integrated analysis of chromatin architecture and FGF2 binding across five chromosomes (chr 13, chr 14, chr 15, chr 21, chr 22) in NC BMSCs. The top panel shows Hi-C contact maps for NC, highlighting chromatin interactions. Below, insulation scores are plotted, with boundary sites indicated by arrows (red for 5’ and blue for 3’). Tracks for super-enhancers (SEs), enhancer sites (ESs), FGF2 binding, FGF2 Hi-Cut binding, H3K27ac, and MED1 occupancy are shown. The bottom panels display FGF2 Hi-Cut contact maps for NC and 1,6-HD treated conditions, along with bar charts comparing insulation scores between NC, OB, and aged groups. This chart illustrates the relationship between FGF2 binding, chromatin insulation, and super-enhancer activity across different genomic regions

To explore the involvement of phase separation FGF2 in the chromosome architecture of the rDNA regions, we further conducted FGF2 related Hi-C & CUT&TAG (Hi-CUT) experiments in normal and phase separation disrupted (treated with 1,6-HD) BMSCs (Figs. 6a, S6A). In line with CUT&TAG, all Hi-CUT results showed that large proportion of FGF2 binding in distal intergenic regions (Fig. S6B). Based on the significant interactions among the conserved proximal centromeric rDNA regions of chromosomes 13, 14, 15, 21, and 22 in Hi-C analysis, we validated that rDNA, and nearby regions were specifically enriched with FGF2 related signals in Hi-CUT (Fig. 6b). By APA analysis, the overlapping signal intensity of Hi-C loops and Hi-CUT loops was markedly higher than Hi-C loops alone, suggesting that FGF2 intervention significantly enhanced the specific looping related interactions, while the interactions diminished after the disruption of phase separation (Fig. 6c). However, compared with normal BMSCs, phase separation disrupted BMSCs related rDNA loop counts were significant decreased (Fig. 6d). Furthermore, combined Hi-C and FGF2 Hi-CUT contact maps, we identified the FGF2 regulated rDNA region and the active interactions region accurate distributed FGF2 related SEs and Es (Fig. 6e). In which, phase separation disruption directly weakens the interaction of rDNA regions, this is consistent with the characterization of FGF2-associated SEs in aged BMSCs (Figs. 6e, 4e, f). Importantly, decreased insulation scores of rDNA region related TAD boundary in aged BMSCs revealed TADs border become insurmountable (Figs. 6e, S6C), which disturbed SEs-mediated TAD fusion and efficient transcription of rDNA. These results indicate that FGF2 and related SEs regulated rRNA expression by modulating rDNA loop and TAD architecture in aged and phase separation BMSCs.

The centromere is the locus where the two sister chromatids of a metaphase chromosome converge, positioned at the primary constriction of the chromosome. It partitions the chromatids into a short arm (p) and a long arm (q) and is constituted of highly repetitive heterochromatin, predominantly composed of DNA and proteins.^48^ Humans possess five such acrocentric chromosomes, located at the short arms of chromosomes 13, 14, 15, 21, and 22, as well as the satellite regions interspersed among them.^49^ Importantly, these regions harbor the invaluable rRNA genes and involve ribosome biogenesis. To scan our findings in global and local perspectives, we integrated and observed comprehensive results of BMSCs at the whole genome as well as the rDNA region by IGVs (Fig. S7A, B). Firstly, the ‌ site of A/B compartment, TADs, and loops are conserved during BMSCs differentiation or aging. In which, proximal chromosomes 13, 14, 15, 21, and 22 related rDNA region is the globally main active interaction regions. Interestingly, in combination with FGF2 Hi-CUT and Hi-C, almost all the conservatively genome-wide interaction regions enriched frequently interacting regions (FIREs),^50^ specific FGF2 binding, and abundant FGF2 SEs, especially in the rDNA regions. However, BMSCs in the phase separation state, the overall lost FGF2-related loops are in the global interaction regions. Next, from local perspective of the rDNA region, in line with the differentiated or aged BMSCs, the counts and intensity of common loops or FGF2 related loops are reduced in phase separation BMSCs.

### FGF2 activates rDNA dynamic in coordination with STAT5

**Fig. 7:**
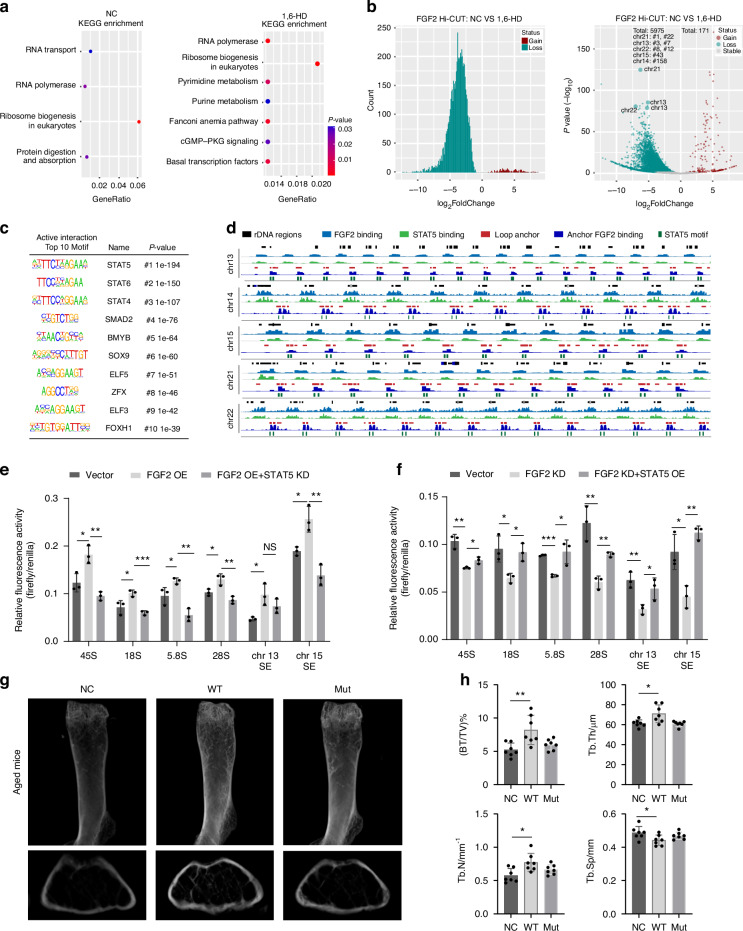
FGF2 activates rDNA dynamic in coordination with STAT5. **a** KEGG pathway enrichment analysis of differentially expressed genes in NC and 1,6-HD-treated BMSCs. The dot plots display the top enriched KEGG pathways. **b** Histogram and volcano plot of FGF2 interaction chromatin loops between NC and 1,6-HD-treated BMSCs. **c** Top 10 enriched motifs associated with active interactions of Hi-C FIREs for normal BMSCs. **d** Genome-wide distribution of FGF2 and STAT5 binding across rDNA regions on chromosomes 13, 14, 15, 21, and 22 in BMSCs. Tracks represent rDNA regions (black), FGF2 binding (blue), STAT5 binding (green), loop anchors (red), and FGF2 binding at loop anchors (dark blue). STAT5 motifs are indicated by green vertical lines. **e**, **f** Relative luciferase activities of containing various rRNA regions (45S, 18S, 5.8S, 28S) and super-enhancer regions (SE1, SE2) reporter plasmid were calculated in response to FGF2 overexpression or knock down in combination with STAT5 knockdown or overexpression. **g** Representative photomicrographs of μ-CT-scanned of distal femurs in aged mice with injection of PBS (NC), wild-type FGF2, and mutation FGF2. **h** Quantitative analysis of μ-CT 3D reconstruction data for bone volume to tissue volume ratio (BV/TV), trabecular number (Tb.N), trabecular thickness (Tb.Th) and trabecular separation (Tb.Sp). *P < 0.05; **P < 0.01; ***P < 0.001; NS *P* > 0.05 by unpaired two-tailed Student’s *t* test

Apart from the consistently global and local manifestation of Hi-C or Hi-CUT in IGV, GO analysis showed FGF2 related loops anchor genes involves nuclease activity and exonuclease activity (Fig. S8A), while the KEGG analysis specific features the pathways related to ribosome biogenesis and RNA polymerase (Fig. 7a). Importantly, the consistency between with FGF2 Hi-CUT and CUT&TAG revealed that FGF2 stable and conservative involved in 3D genome regulation, especially in the rDNA region. Additionally, the reduced loops in the phase separation disruption BMSCs were mainly located in the rDNA region associated with chromosomes 13, 14, 15, 21, and 22 (Figs. 7b, S8B). By global motif identification of Hi-C FIREs and Hi-CUT, we confirmed the high overlap of STAT5, SMAD2, and SOX9 (Fig. 7c). Furthermore, we validated the rDNA related FGF2 loop anchors present a one-to-one correspondence of STAT5 motif in detail, which provides a strong evidence chain for 3D genome regulation of FGF2 (Fig. 7d). Along with the co-binding of FGF2 and STAT5 in rDNA region, we further confirmed the interactions between FGF2 and STAT5 (Fig. S8C).

To further identified the mechanism of FGF2/STAT5 complex regulated SEs related rDNA transcription, we designed the luciferase reporter plasmids and the rescue experiments. After knock-down or overexpression of FGF2, luciferase reporter plasmids target rRNA regions and related SEs showed the decreased or increased activity, respectively (Fig. 7e, f). However, the altered activity was rescued by STAT5 overexpression or knock-down (Fig. 7e, f).

Subsequent in vivo investigations confirmed that tail vein administration of wild-type FGF2 protein significantly enhanced both bone mass and osseous regenerative capacity in senescent mouse models (Fig. 7g, h). Then we evaluated the cranial defect regenerative capacity and the results demonstrate that wild-type FGF2 administration significantly augments cranial defect regeneration in animal models (Fig. S8D). Moreover, H&E and Masson’s trichrome staining of femoral tissues provide direct histological evidence of FGF2-mediated tissue repair and regeneration, including collagen deposition, osteoblast activity, and mineralization patterns (Fig. S8E).

## Discussion

One aspect that has received significant interest over the past decade is the link between the rRNA genes and genome architectures in nucleus, which is crucial for cell fate transition.^51,52^ Previous research proved that nuclear FGF2 accelerated rRNA transcription undergoing cell cycle homeostasis and cell proliferation.^15,53^ Meanwhile, nuclear protein related phase separation involved TADs reorganization and chromatinic looping in impacting special regulatory transcription elements.^54^ However, the mechanisms of FGF2 nuclear transport and genomic regulation of rRNA transcription remain unclear. Here, we demonstrate that the stable NLS motif controls the FGF2 nuclear translocation and phase separation during BMSCs cell fate transition. Furthermore, FGF2 and its phase separation regulates rRNA expression by rDNA associated epigenetic inheritance. Despite the distinction of chromatin architectures during BMSCs differentiation and aging, phase separation FGF2 contribute to rDNA related TAD integration and rRNA transcription by accurate recognition of specific SEs and STAT5 motifs.

As a key growth factor, FGF2 is widely acknowledged for its role in maintaining BMSCs pluripotency and self-renewal.^55–57^ In line with previous findings, our study reveals that FGF2 is intricately linked to cell cycle regulation and proliferation. Notably, the phosphorylation of specific NLS motif in FGF2 enhances multimer formation, destructs phase separation and the creation of condensates. This process notably reduces its interaction with nuclear transport machinery, thereby disrupting nuclear translocation. This disruption is critical, as phase separation underpins the organization of nuclear compartments and facilitates functional chromatin interactions essential for gene transcription regulation.^58–60^ The global disruption of cellular condensates by 1,6-hexanediol further validates that FGF2 assemblies are indeed driven by liquid-liquid phase separation. In vitro studies of FGF2 mutants with strategically altered phosphorylation sites confirm that these phase transitions directly link FGF2 condensation behavior to its regulatory role in rRNA transcription. Furthermore, FGF2 interaction with super-enhancers in the rDNA region is pivotal for sustaining high rRNA transcription levels, as these super-enhancers act as regulatory hubs within the nucleolus. Our findings indicate that reduced FGF2 enrichment at the rDNA regions of centromeric chromosomes, observed during differentiation and aging, corresponds with diminished rRNA transcription. This establishes a mechanistic connection between FGF2-mediated phase separation, super-enhancer activity, and rRNA expression, highlighting FGF2 as a key molecular nexus linking chromatin architecture with transcriptional regulation in specialized nuclear domains.

Higher-order chromatin structures, such as disruptions in TAD boundaries and looping anchors, are increasingly recognized as pivotal regulators of gene expression and cell fate decisions.^61–63^ Pre-establishing key TAD architectures characteristic of late-stage cells within early-stage cells may facilitate cell fate transitions.^64,65^ Through mapping genome-wide chromatin interactions during BMSCs differentiation and aging, we identified dynamic reorganizations of higher-order chromatin structures across multiple hierarchical levels in chromosomes 13, 14, 15, 21, and 22 related proximal centromere that house the rDNA region. Notably, interactions within these centromeric rDNA regions weakened during BMSCs differentiation and senescence, accompanied by TAD reorganization. This reorganization likely corresponds to the redistribution of super-enhancers within TADs, thereby altering their target loci. These modifications represent broader reprogramming processes as BMSCs transition from a proliferative state to terminal differentiation or aging. The alterations observed in the rDNA regions suggest that these topological shifts are intricately linked to the functional demands of ribosomal biogenesis, reflecting a strategic reallocation of cellular resources as BMSCs lose their proliferative capacity.

Our findings reveal that FGF2 plays a central role in orchestrating 3D genome reorganization during BMSCs fate transitions. FGF2 enhances chromosomal interactions, particularly in the rDNA region, where TAD boundaries are enriched with FGF2-linked super-enhancers (SEs) and active enhancer signals. During aging progress, increasing isolation of TAD boundaries limits the influence of adjacent FGF2-SEs, suggesting that TAD remodeling directly regulates FGF2-driven rRNA transcription. This spatial reorganization is mediated by FGF2 interaction with nuclear matrix-associated protein complexes that modulate enhancer activity and chromatin accessibility. Moreover, FGF2 forms dynamic nuclear condensates that colocalize with SEs enriched in STAT5 binding sites, potentially facilitating STAT5 activation at lineage-specific regulatory loci. The synergistic interaction between FGF2 and STAT5 underscores a coordinated mechanism linking chromatin architecture to transcriptional control during differentiation and in response to metabolic stress.

Together, our study delineates the dual role of FGF2 as both an extracellular growth factor and a nuclear regulator of chromatin architecture. By elucidating the molecular basis of FGF2 nuclear translocation and its capacity to drive phase separation at super-enhancer loci, we uncover a mechanism for spatially confined transcriptional control during cell fate transitions. These findings offer a conceptual framework for harnessing nuclear-targeted delivery of bioactive molecules, paving the way for precision modulation of gene expression in regenerative medicine and age-related tissue remodeling.

## Materials and methods

### Animals

Animal experimental procedures were approved by the Laboratory Animal Care and Use Committee at Anhui Medical University (Approval No. LLSC20220738). The 3-month-old male C57BL/6 mice serve as the normal group, while the 18-month-old male C57BL/6 mice serve as the aged group. The sample size (*n* = 7) and inclusion criteria of each group was confirmed with adequate power based on the literature and our previous experience^66^. The mice were euthanized, and the alveolar bone were harvested for histological analysis.

### Primary cell culture

For the isolation of BMSCs from human alveolar bone, volunteers aged 18–32 (normal) and 62–79 (aged) with provided informed consent, participated in this study. The inclusion criteria and cell culture protocols followed established methods as described in reference^66^. All procedures involving human BMSCs were approved by the Ethical Committee Department at the College & Hospital of Stomatology of Anhui Medical University (Approval No. T2021014). Regarding mouse BMSCs isolation, the bone marrow was flushed with α-MEM using an 18-gauge sterile needle inserted into the medullary cavity. The following protocols were performed in accordance with human BMSCs culture. Normal and aged BMSCs were grown on Gibco™ BASIC MEM α (Thermo Fisher) containing 10% Fetal Bovine Serum (ScienCell) and 1% penicillin/streptomycin and maintained at 37 °C in 5% CO2. HEK293T cells were cultured in DMEM (Solarbio) containing 10% Fetal Bovine Serum (Prime) (ExCell Bio) and 1% penicillin /streptomycin.

### Single cell RNA seq

Single-cell RNA-seq raw data generated in telomerase-immortalized human BMSCs at day 0 and day 7 of osteogenic induction were retrieved from NCBI Gene Expression Omnibus (GEO), labeling GSM3439738 and GSM3439737. Sequence Read Archive (SRA) raw data were downloaded from NCBI servers. In total, the data included 2 221 undifferentiated cells, and 3 329 cells after 7 days of osteogenic induction. Quality controls and global-scaling normalization were performed in Seurat. The top 2000 feature genes were scaled for follow-up principal component analysis (PCA) reduction. According to JackStrawPlot and ElbowPlot, principal components were selected for tSNE and UMAP cluster analysis. Next, differentially expressed genes (DEGs) of each cluster were found by Seurat and tested using receiver operating characteristic (ROC) curves. Based on the Seurat process, the predicted differentiation score was calculated by CytoTRACE.

### IP

In total, 2–5 × 10^7^ BMSCs were lysed on the culture dish in 1.0 mL ice cold RIPA buffer (Beyotime). The lysates were incubated for 30 min on ice while shaking, followed by a centrifugation at 13 000 r/min for 15 min at 4 °C. The supernatant was used in immunoprecipitation reactions using the Protein A/G PLUS-Agarose (Santa Cruz Biotechnology) according to the manufacturer’s instructions. Briefly, transfer 1 mL of the above cell lysates to a 1.5 mL microcentrifuge tube. Add 20 µL (1:50) FGF2 antibody (Abcam) and incubate for 2–4 h at 4 °C. Preclear lysate by adding 1.0 µg of the control IgG (proteintech) and incubate for 2–4 h at 4 °C. Add 40 µL of resuspended volume of Protein A/G PLUS-Agarose. Cap tubes and incubate at 4 °C on a rocker platform for overnight. On the next day, the beads-antibody-protein complexes were washed 3 times with washing buffer, re-suspended pellet in 2 ×SDS-PAGE Protein Sample Loading Buffer (Beyotime) and incubated at 95 °C for 10 min. The samples were separated on a 10% SDS-PAGE used for immunoblotting analysis. Remaining samples were subjected to whole lane LC-MS/MS sequencing and data analysis. Three biological replicates were performed for the identification.

### LC-MS/MS

Digestion of sample protein was performed according to the FASP procedure^67^. Briefly, the detergent, DTT and other low-molecular-weight components were removed using 200 μL UA buffer (8 mol/L Urea, 150 mmol/L Tris-HCl pH 8.0) by repeated ultrafiltration (Microcon units, 10 kD) facilitated by centrifugation. Then 100 μL 0.05 mol/L iodoacetamide in UA buffer was added to block reduced cysteine residues and the samples were incubated for 20 min in darkness. The filter was washed with 100 μL UA buffer three times and then 100 μL 25 mmol/L NH_4_HCO_3_ twice. Finally, the protein suspension was digested with 3 μg trypsin (Promega) in 40 μL 25 mmol/L NH_4_HCO_3_ overnight at 37 °C, and the resulting peptides were collected as a filtrate. Experiments were performed on a Q Exactive HF-X mass spectrometer that was coupled to Easy nLC (Thermo Fisher Scientific). The peptide mixture was loaded onto a the C18-reversed phase column (15 cm long, 75 μm inner diameter) packed in-house with RP-C18 5 μm resin in buffer A (0.1% Formic acid in HPLC-grade water) and separated with a linear gradient of buffer B (0.1% Formic acid in 84% acetonitrile) at a flow rate of 250 nL/min controlled by IntelliFlow technology over 60 min. MS data was acquired using a data-dependent top10 method dynamically choosing the most abundant precursor ions from the survey scan (300–1 800 m/z) for HCD fragmentation. Determination of the target value is based on predictive Automatic Gain Control (pAGC). Dynamic exclusion duration was 20 s. Survey scans were acquired at a resolution of 70 000 at m/z 200 and resolution for HCD spectra was set to 17 500 at m/z 200. Normalized collision energy was 27 eV and the underfill ratio, which specifies the minimum percentage of the target value likely to be reached at maximum fill time, was defined as 0.1%.

The MS data were analyzed using MaxQuant software version 1.3.0.5. MS data were searched against the UniProtKB Homo sapiens database (194237 total entries, downloaded 20201210). An initial search was set at a precursor mass window of 6 × 10^6^. The search followed an enzymatic cleavage rule of Trypsin and allowed maximal two missed cleavage sites and a mass tolerance of 20 × 10^−6^ for fragment ions. carbamidomethyl (C) was defined as fixed modification, while oxidation (M), phosphorylation (S/T/Y) was defined as variable modifications for database searching. The cutoff of global false discovery rate (FDR) for peptide and protein identification was set to 0.01.

### HiC

The HiC experiments were performed by Wuhan Frasergen Bioinformatics Co., Ltd. Initially, 5–10 × 10^7^ cells were cross-linked with 1% formaldehyde for 10 min at room temperature. Cells were lysed to isolate nuclei using a hypotonic buffer containing 0.2% Igepal CA630. Chromatin was digested with 20 U MboI (NEB) at 37 °C overnight. For biotinylation, 37.5 μL biotin-14-dATP (Life Technologies) and 8 U Klenow fragment (NEB) were added, then incubated at 37 °C for 4 h. Ligation was performed using 5 U T4 DNA ligase (NEB) at room temperature for at least 4 h. Then the DNA was decrosslinked, purified, and fragmented into approximately 400 bp fragments, after which streptavidin-coated magnetic beads were utilized to enrich ligation junction fragments for subsequent Illumina library preparation and sequencing. In situ Hi-C was performed as previous research^23^.

After quality filtering using Trimmomatic^68^, the clean Hi-C data of three samples with two biological replicates for each group, was mapped to the genome by using the BWA mem. Then, PCR duplicates were filtered by pairtools. Above process reference Hi-C pipeline (https://www.encodeproject.org/hic/). The valid pairs were used to analyze the correlation efficiency of the two biological replicates for each sample using GenomeDISCO software,^69^ then we pooled the data from two replicates together for resolution enhancement and further analysis. The valid pairs after pooling were binned into 200 kb nonoverlapping genomic intervals to draw the interaction heatmap and calculate contact decay curve.

The cooltools expected-cis software^70^ was applied to identify A and B compartments on 200 kb resolution. The locations of TADs can be determined when interaction data is binned at 40 kb. We used the cooltools insulation score algorithm^71^ to identify the locations of TAD boundaries of each sample.

We first use mustache to detect loops at 5 kb-10 kb-20 kb resolution. Then, we combined all loops at different resolutions. If an interaction was detected as a loop at different resolutions, we retained the precise coordinates in finer resolutions and discarded the coarser resolution.^72^

### CUT&Tag

The Hyperactive Universal CUT&Tag Assay Kit for Illumina Pro (Vazyme) was used for CUT&Tag assay according to the manufacturer’s instructions. In brief, ConA Beads Pro were activated in Binding Buffer, washed, and resuspended. Cells (5–10 × 10⁴) were centrifuged, washed, and bound to activated beads. After incubation, cells were resuspended in Antibody Buffer with primary antibodies and incubated overnight at 4 °C. Secondary antibody (1:100 in Dig-wash Buffer) was added and incubated for 30–60 min at room temperature. Samples were washed twice with Dig-wash Buffer, then treated with pA/G-Tnp Pro in Dig-300 Buffer for 1 h. Following two additional washes, transposase reaction was initiated in 5×TTBL/Dig-300 Buffer at 37 °C for 60 min. DNA was released using 10% SDS and DNA Spike-in at 55 °C, then purified using DNA Extract Beads Pro with B&W Buffer washes. Purified DNA was amplified via PCR (9-12 cycles) using index primers (N5XX/N7XX) and CAM master mix. PCR products were cleaned with VAHTS DNA Clean Beads (Vazyme #N411), eluted in ddH₂O, and prepared for sequencing quality control. Detailed information can be found in the article.^73^

After obtaining clean data, we used Bowtie2^74^ to map clean reads to the reference T2T genome (information: https://www.ncbi.nlm.nih.gov/data-hub/genome/GCF_009914755.1/, download from: https://ftp.ncbi.nlm.nih.gov/genomes/all/GCF/009/914/755/GCF_009914755.1_T2T-CHM13v2.0/), and further screen out and removed the PCR duplicates, low-quality alignment, and organelle alignment by Picard^75^, and thus to get the retained valid pairs for subsequent analysis. Next, we choose HOMER^76^ software based on statistical methods to perform Peak Callin. After that, the R scripts with Deeptools software^77^ were performed to illustrate the signal enrichment analysis of transcription start site (TSS), gene body and peak regions. Finally, we randomly selected the enrichment regions on genome, and visualized the called peaks of all samples using the Integrative Genomic Viewer (IGV).^78^

DiffBind^79^ was utilized to detect potential differential peaks (DP) from peak sets. Statistically significant DP were defined as peaks with |log_2_FoldChange | >0.58 and FDR < 0.05, and the DP signal of each sample between groups was clustered for analysis. ChIPseeker^80^ was used to annotate the DP to the genome and to find the genes associated with DP (Peaks are annotated based on their mapping to the nearest transcription start site). And the DP associated genes were used to perform GO and KEGG analyses using ClusterProfiler. Motif analysis on DP was performed by HOMER^76^ suite with default settings.

FGF2 Cut&Tag analyses were performed on human BMSCs during osteoblast differentiation and aging. HOMER find-peaks were run on FGF2 Cut&Tag with augment ‘-style histone -nfr -size 1 000' to detect peaks with specificity for histones and nucleosome free regions (Poisson *P* value threshold = 0.001). Rank Ordering of SEs (ROSE) was utilized to identify and analyze SEs. All visualization tasks were managed using Integrative Genomics Viewer (IGV).

### Hi-CuT

The HiCuT experiments were conducted by Wuhan Frasergen Bioinformatics Co., Ltd., building on previous methodologies^73,81^ with some modifications. Initially, samples were primary cross-linked using 1% formaldehyde for 10 min at room temperature and then secondary crosslinking with 3 mmol/L EGS for 40 min. The cells are mildly permeabilized with 0.05% digitonin. Chromatin DNA was digested with 50U MboI and 50U DdeI (NEB) at 37 °C for 2 h without biotinylation. Spatial proximal DNA ligation was achieved using 50U T4 DNA ligase (NEB) at 16 °C for 4 h. The part of HiC was performed as previous.^82^ After being washed twice, Cells was used for the part of CUT&Tag. CUT&Tag have been described in detail.^81^

### RNA isolation and qRT-PCR

Total RNA was extracted from cell pellets using RNAiso Plus (Taraka) and cDNA was synthesized using HiScript II QRT SuperMix for qPCR (Vazyme). Real time qPCR was performed using AceQ qPCR SYBR green Master Mix (Vazyme) on CFX96 Touch Real-Time PCR Detection System (USA). The exhibited data in the paper represents the relative fold change of experimental group versus control group. Briefly, ΔCt was calculated as ΔCt = Ct (test gene) – Ct (housekeeping gene). ΔΔCt was calculated as ΔΔCt =ΔCt (experimental group) -ΔCt (control group). The relative fold change of a test gene in experimental group versus control group was calculated as 2^−ΔΔCT^. Each gene tested in triplicates in independent experiment. Primers used for qPCR are listed in Table S1.

### Protein extraction and western blots

Cells were lysed on the culture dish in RIPA Lysis Buffer (Beyotime). The lysates were incubated for 20 min on ice while shaking, followed by a centrifugation at 13 000 r/min for 10 min. The supernatant was moved into a new 1.5 mL microcentrifuge tube, added 2 ×SDS-PAGE Protein Sample Loading Buffer (Beyotime), then boiled at 95 °C for 10 min, and placed in –20 storage or directly for western blots detection. Protein samples were separated on 10% Bis-Tris gel, and then wet transferred to a 0.45 μm PVDF membrane (Bio-Rad) in ice-cold transfer buffer for 2 h. After blocked with 8% non-fat milk for 1 h at room temperature, the membrane was incubated with the primary antibody overnight at 4 °C. After washed four times with TBST for 7 min, the membrane was incubated with HRP-conjugated secondary antibodies for 1 h at room temperature. After washed four times with TBST. The membrane was imaged using a CCD camera for visualizing the immunoreactive bands using enhanced chemiluminescence detection (Tanon, Shanghai, China). All key resources are listed in Table S2.

### CCK8 assays

In total, 200 μL BMSCs cells suspension was inoculated on 96-well plate and incubated in cell incubator (37 °C, 5% C02). Add 20 µL volume of Cell Counting Kit-8(CCK-8) directly to cells in culture medium. Mix thoroughly to achieve a homogenous solution. Incubate in a cell culture incubator for 1 h at 37 °C until the color turns orange. Place the 96-well plate on the shaking table for about 1 min before the reading of the micrometer. The 450 nm light absorption value was read by an enzyme marker and cell activity was calculated.

### Immunofluorescence

Add a drop of complete culture medium to a six-well plate, then place a coverslip inside. Add 500 µL of cell suspension at the appropriate concentration onto the coverslip. Incubate the assembly in a culture incubator for 4 h, then add 2 mL of culture medium and continue the incubation overnight. On the following day, cells cultured on the coverslip were fixed with 4% paraformaldehyde for 20 min, followed by three washes with PBS. Cells were then permeabilized using 0.3% Triton X-100 for 20 min. After another three PBS washes, the cells were blocked with 5% BSA at 37 °C for 30 min before overnight incubation at 4 °C with primary antibodies. Following three additional PBS washes, the cells were incubated with corresponding secondary antibodies for 1 h at room temperature without light exposure. After three PBS washes, the cell nuclei were stained with DAPI (1 µg/mL) for 4 min. A drop of Antifade Mounting Medium (LEAGENE) was then added to the slide. The coverslip was carefully removed, inverted onto the slide, and fluorescent images were captured using microscopy.

### Preparation of FGF2 labeled with rhodamine B (RhB-FGF2)

Rhodamine B isothiocyanate (5 mg) was dissolved in DMSO (1 mL) and then added to the recombinant FGF2 solution (0.1 mol/L Na_2_CO_3_, 50 mg/mL, 2 mL). The mixture was stirred at room temperature for 2 h before dialysis in deionized water (3 × 4 L). After lyophilization, purple RhB-FGF2 was obtained. All key resources are listed in Table S2.

### FRAP

BMSCs were cultured in confocal dishes (Corning Incorporated, Corning, NY, USA), and RhB-FGF2 (50 ng/mL) was added for a 24-h incubation. Fluorescence images of RhB-FGF2 were acquired on a ZEISS LSM 980 confocal microscope using a ×100 oil-immersion objective lens. The fluorescence intensity of bleached cell at each time point was normalized by fluorescence intensity at background region and fluorescence intensity of adjacent unbleached cell. The images were analyzed using Fiji - ImageJ software.

### Sedimentation assays

Recombinant FGF2 was mixed with the same amount of PBS with 0% or 20% PEG and samples were centrifuged at 2 500 × *g* for 5 min. The supernatant was collected in a new tube and the pellet was resuspended in PBS. Supernatant and resuspended FGF2 were collected by adding 5× Non-denatured Gel Protein Sample Loading Buffer (Beyotime) and subsequently resolved by SDS–PAGE. The gel was immediately stained with Coomassie staining solution (Beyotime) for 30 min and then soaked in distilled water until the band is clear and the background transparent.

### Lentiviral production for RNA interference and gene overexpression

HEK293T cells were used to produce lentiviral particles. Lentivirus for gene knockdown was generated in HEK293T cells by co-transfection with pLKO.1-shRNA, pRSVRev, gag/pol, and pVSV-G at the ratio of 2:2:2:1with transfection reagent Lipofectamine 2000 (Thermo Fisher). Targeting sequences of RNA interference are shown in Table S1. Similarly, lentivirus for gene overexpression was generated in HEK293T cells by co-transfection with pCDH-FGF2, psPAX2, and pMD2.G at the ratio of 2:1:5:1. 6–8 h after transfection, the culture medium was refreshed with a new supply. Supernatant containing lentivirus was harvested after 48 h transfection and filtered through 0.45 μm filters before using for infecting target cells. After 24 h infection, cells were treated with 5 mg/mL puromycin (Beyotime) for 48 h to select successfully transduced cells.

### Statistical analyses

All data were expressed as mean ± SD. Unpaired two-tailed Student’s *t* test was used for comparison between two groups. Data between multiple groups were compared with one-way analysis of variance (ANOVA) followed by the Tukey’s post hoc test. *P* < 0.05 was considered as statistically significant. GraphPad Prism 9 software was used for data analysis and figure generation.

## Supplementary information

Supplementary Figures

Supplementary Tables

## Data Availability

Cut&Tag, Hi-C, and Hi-Cut data were uploaded at China National Center for Bioinformation / Beijing Institute of Genomics, Chinese Academy of Sciences (BioProject: PRJCA029726, https://ngdc.cncb.ac.cn/gsa). The reference genome is T2T genome (versions: human_T2T_CHM13v2.0). Mass spectrometric data were uploaded at iProX (Project ID: IPX0009728000).
