# Supplementary material for: Nuclear FGF2 orchestrates phase separation-mediated rDNA chromatin architecture to control BMSCs cell fate
Source: Bone Res. 2025 Sep 24;13:80. doi: 10.1038/s41413-025-00451-y (PMC12460815; doi:10.1038/s41413-025-00451-y)
Supplement: Supplementary file 1 — Supplementary Figures [file 41413_2025_451_MOESM1_ESM.docx]

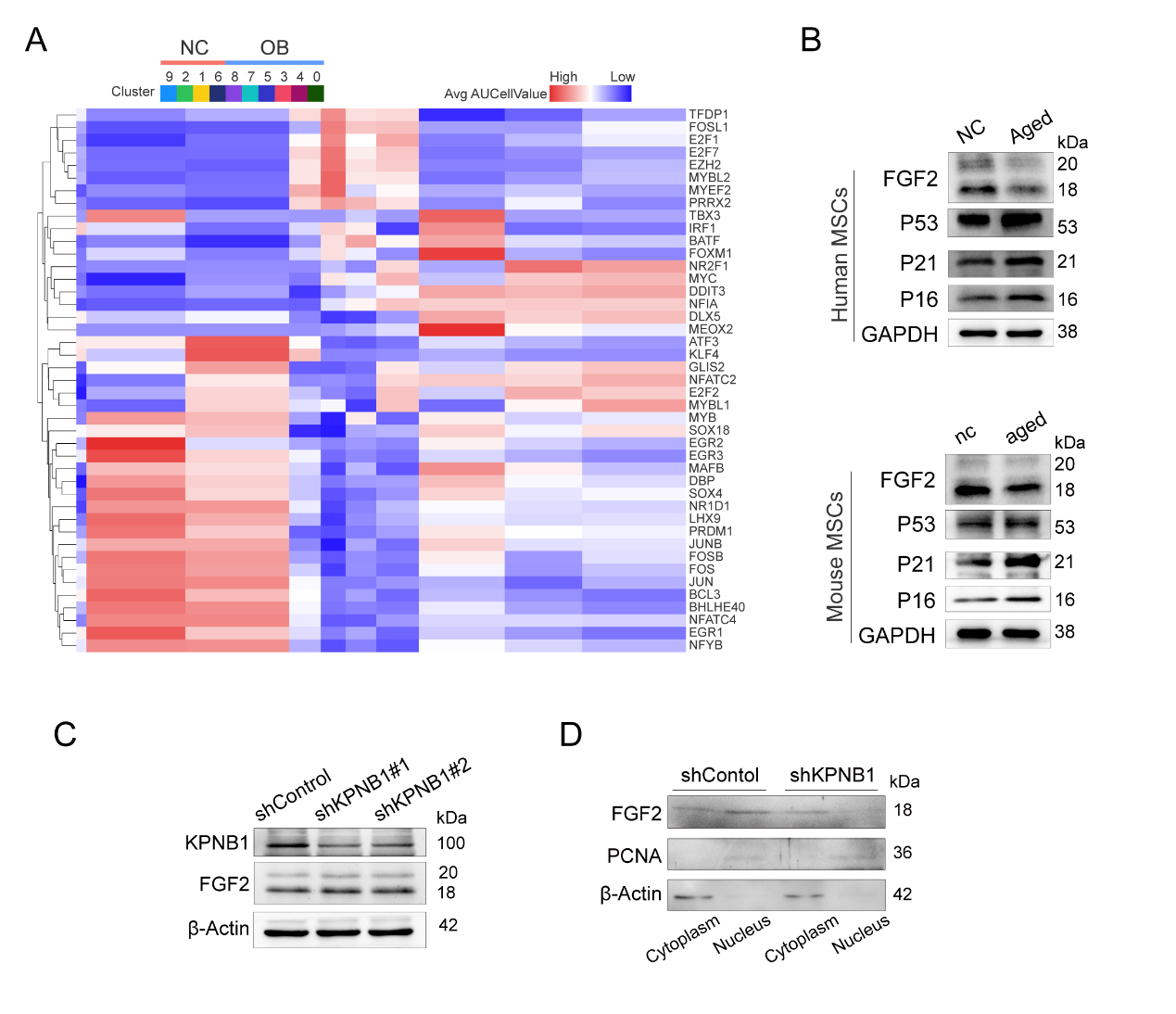


**Figure S1. FGF2 and its nuclear translocation mediates MSCs cell fate, related to Figure 1**

(A) Heatmap showing the average AUCell values for key transcription factors across NC and OB MSCs. Each row represents a different transcription factor, with clustering performed to group similar expression patterns. (B) Western blotting against FGF2, P53, P21, and P16 in normal and aged human MSCs (upper panel), and normal and aged mouse MSCs (lower panel). (C) Western blot analysis showing the effects of KPNB1 knockdown on FGF2 expression. The panel displays the protein levels of FGF2 in whole-cell lysates from cells transfected with shControl, shKPNB1#1 and shKPNB1#2. (D) The panel shows the subcellular localization of FGF2 in the cytoplasmic and nuclear fractions after KPNB1 knockdown.


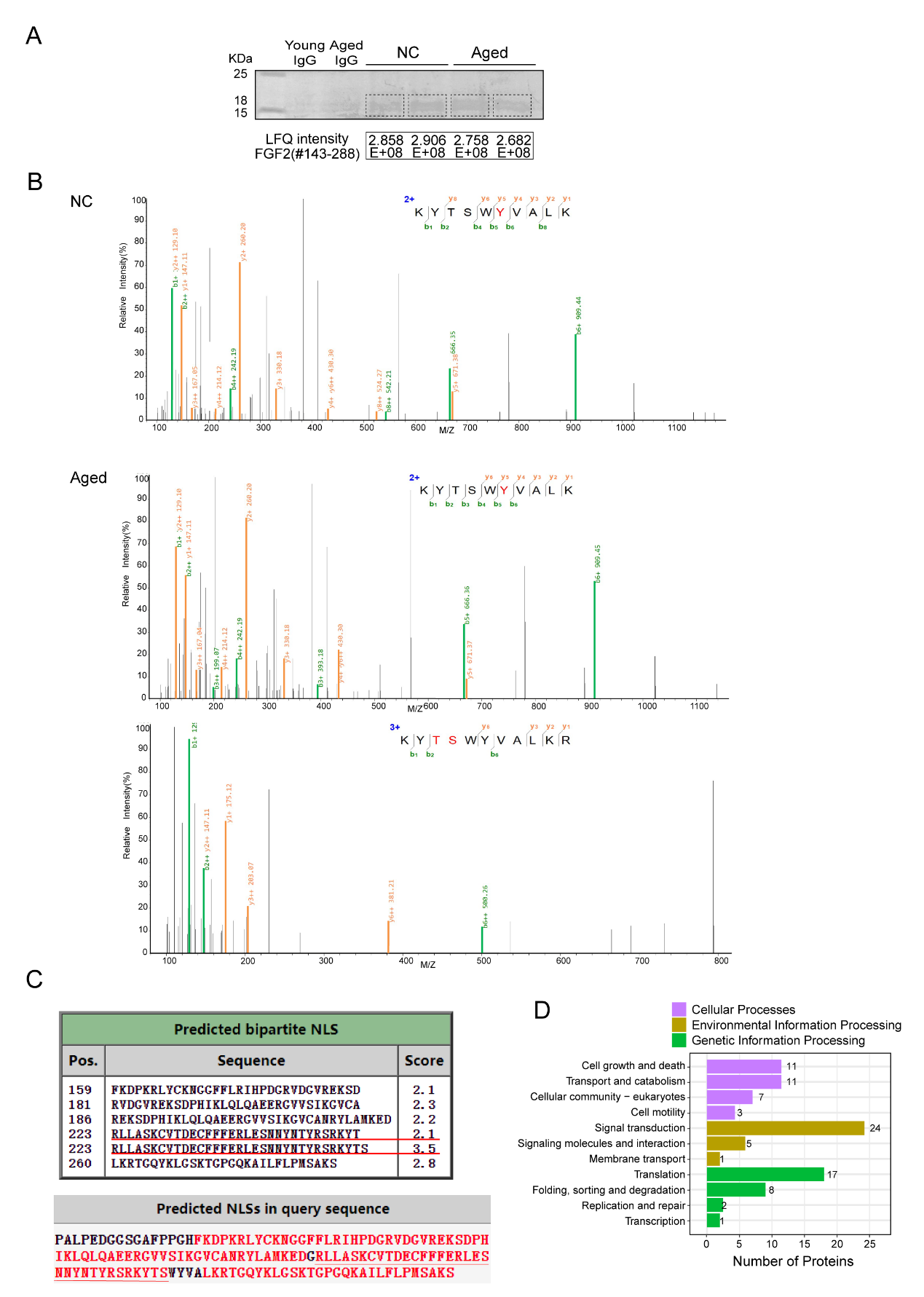


**Figure S2. Nuclear FGF2 regulates MSCs rRNA synthesis, related to Figure 2**

(A-B) Mass spectrometry analysis of FGF2 phosphorylation in NC and aged MSCs. The spectra show the relative intensity of detected ions across different mass-to-charge (m/z) ratios for a specific phosphorylated peptide (KYTTSWYVALK) in both conditions. (C) Predicted bipartite nuclear localization signals (NLS) within the FGF2 protein sequence. The table lists the position (Pos.), sequence, and corresponding score for each predicted NLS. The sequence with the highest score (3.5) is highlighted, indicating a strong likelihood of functioning as an NLS. (D) Gene Ontology functional annotation analysis for biological processes occupied by FGF2.


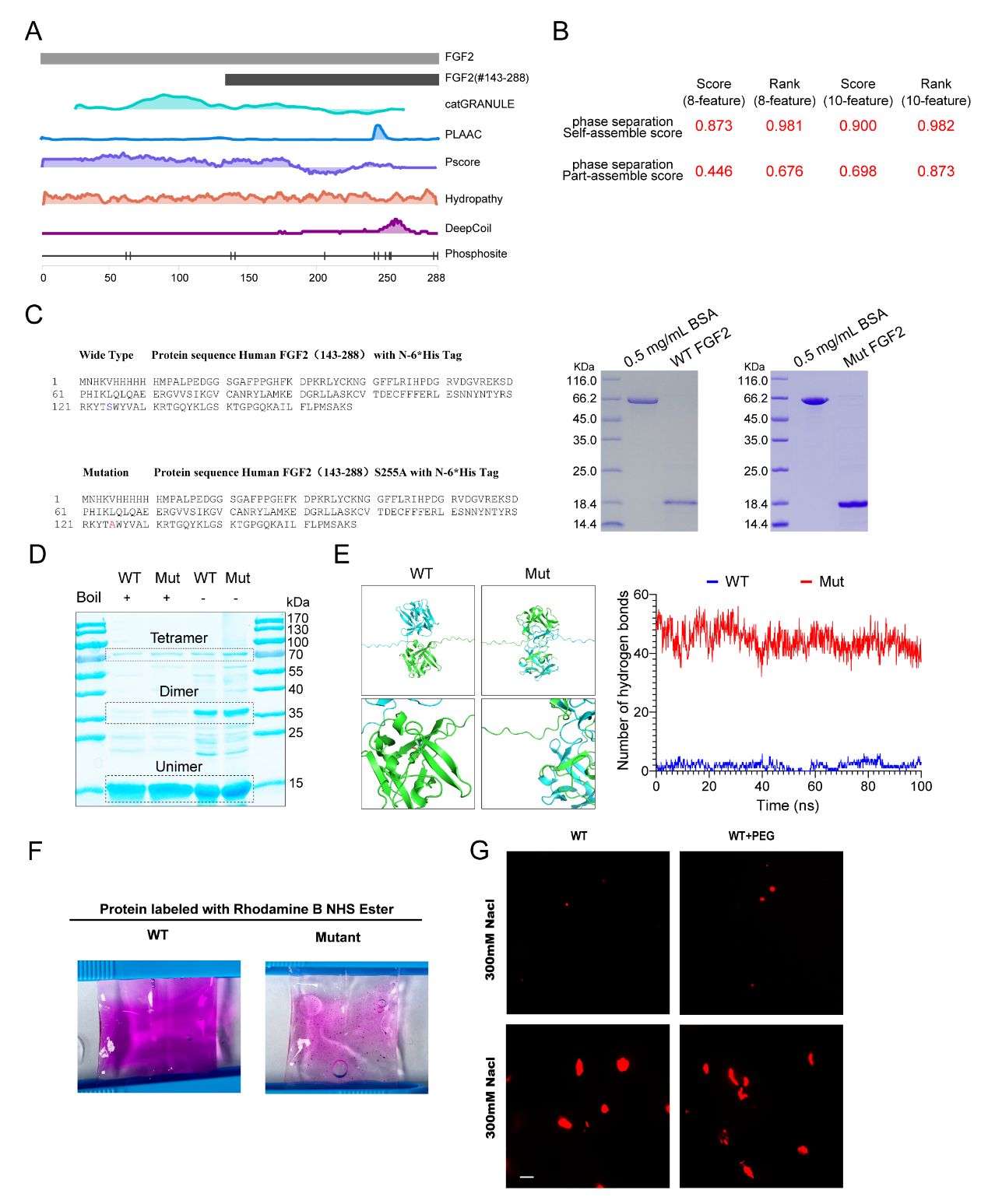


**Figure S3. FGF2 regulates rRNA synthesis via phase separation, related to Figure 3**

(A) Domain and feature analysis of the FGF2 protein sequence. The top bars represent the full FGF2 protein, and the specific region (residues 143-288) analyzed. The plots below show various computational predictions for this region, including catGRANULE (predicting phase separation potential), PLAAC (predicting prion-like domains), Pscore (predicting disorder propensity), Hydropathy (indicating hydrophobicity), DeepCoil (predicting coiled-coil regions), and Phosphosite (indicating known phosphorylation sites). (B) Phase separation analysis of FGF2 using different feature sets. The table presents the scores and ranks for self-assembly and part-assembly potential in phase separation, evaluated using 8-feature and 10-feature models. (C) Amino acid sequences of wild-type and S255A mutant FGF2 (residues 143-288) with an N-terminal 6xHis tag (left). The wild-type sequence is shown at the top, while the S255A mutant sequence, where serine at position 255 is replaced by alanine, is shown at the bottom. Both WT and mutant FGF2 were expressed and purified, with 0.5 mg/mL BSA used as a loading control (right). (D) Representative Coomassie brilliant blue (CBB) staining of wild-type-FGF2 and mutant-FGF2 fractions after 10 minutes of denaturation at 95°C, alongside samples that were not denatured. Unimer (~ 18 kDa) and estimated Dimer (~ 35 kDa) and Tetramer FGF2 (~ 70 kDa) can be observed. (E) Structural predictions for the wild-type-FGF2 and mutant-FGF2 by AlphaFold2, left panel. The number of hydrogen bonds formed by wild-type-FGF2 and mutant-FGF2 was tracked over time, right panel. (F) Biophysical characterization of WT- and Mut-FGF2 post-rhodamine conjugation and dialysis. (G) In vitro droplet formation assays of WT- and Mut-FGF2 post-rhodamine conjugation and dialysis. Scale bars, 2 μm.


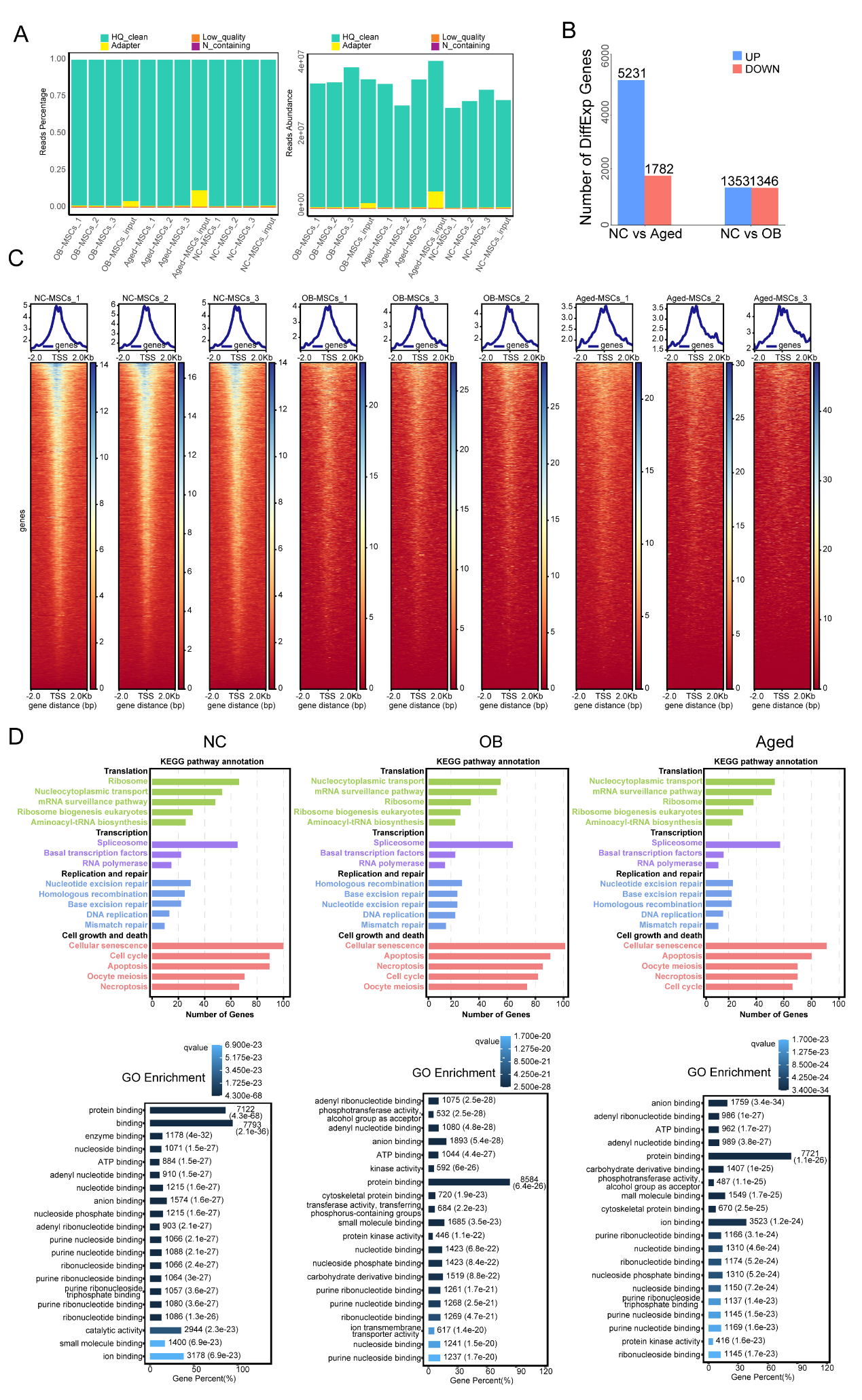


**Figure S4. FGF2 involves in rDNA regions related epigenetic regulation, related to Figure 4.**

(A) Quality control (QC) analysis of CUT&TAG sequencing reads from NC, OB, and aged MSCs. (B) Bar chart showing the number of differentially expressed (DiffExp) genes between NC and aged MSCs, and between NC and OB MSCs. (C) Heatmaps and average profiles showing the distribution of CUT&TAG signal intensity around transcription start sites (TSS) for genes in NC, OB, and aged MSCs samples. (D) KEGG pathway annotation and Gene Ontology (GO) enrichment analysis of genes interacting with FGF2 in normal MSCs, based on three replicates of data.


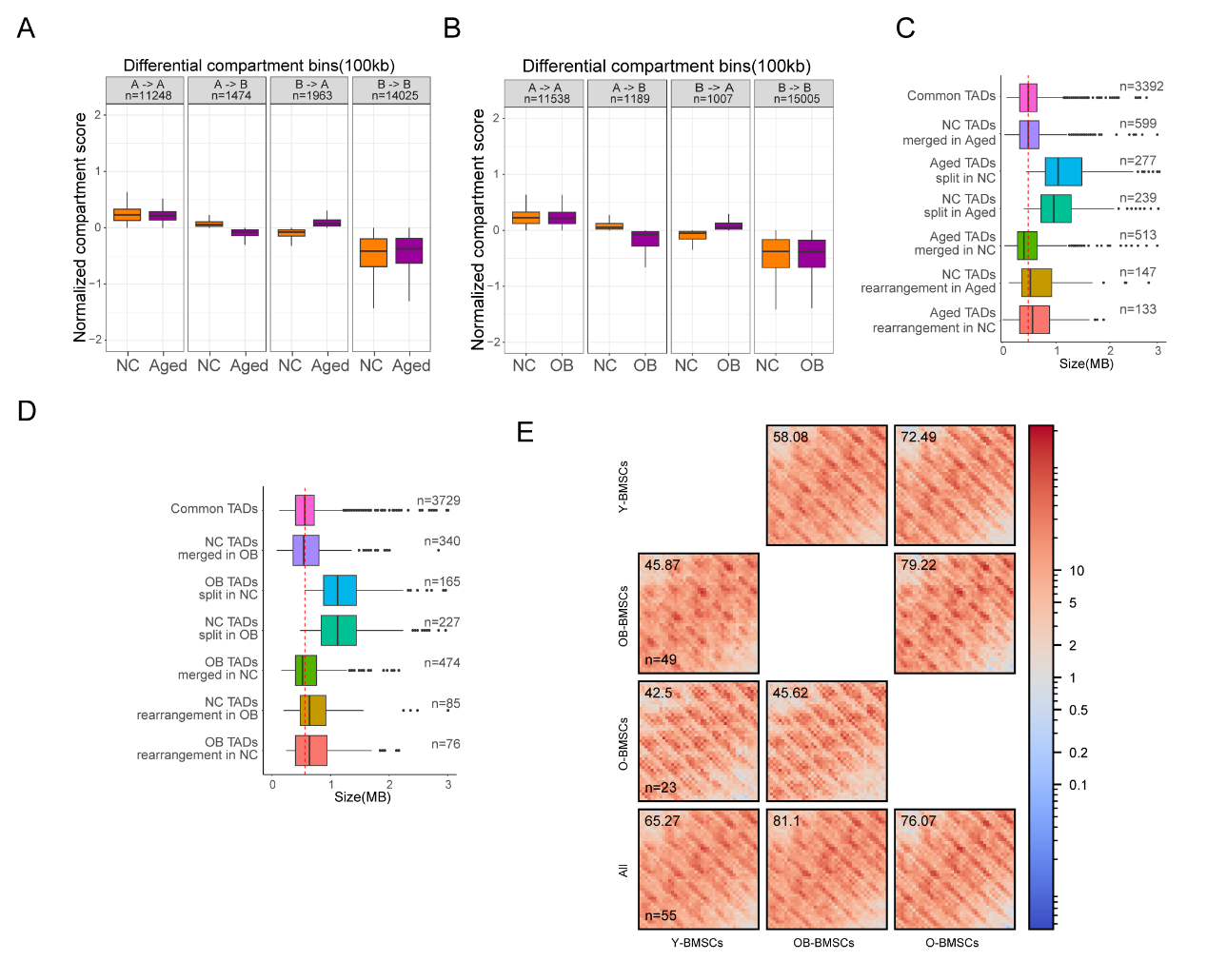


**Figure S5. Chromatin architectures feature of rDNA regions during MSCs cell fate transition, related to Figure 5**

(A-B) Box plots showing the normalized compartment scores for differential compartment bins (100 kb) between NC and aged MSCs, and between NC and OB MSCs. The box plots illustrate the changes in chromatin compartmentalization associated with aging, with shifts between active A and inactive B compartments. (C) Box plots showing the size distribution of Topologically Associating Domains (TADs) in various categories between NC and aged MSCs, and between NC and OB MSCs. Highlighting the structural dynamics of TADs associated with aging, including the merging, splitting, and rearrangement of TADs. (E) Heatmaps showing the enrichment of Mustache-detected loops in NC, OB, aged groups, and merged all groups.


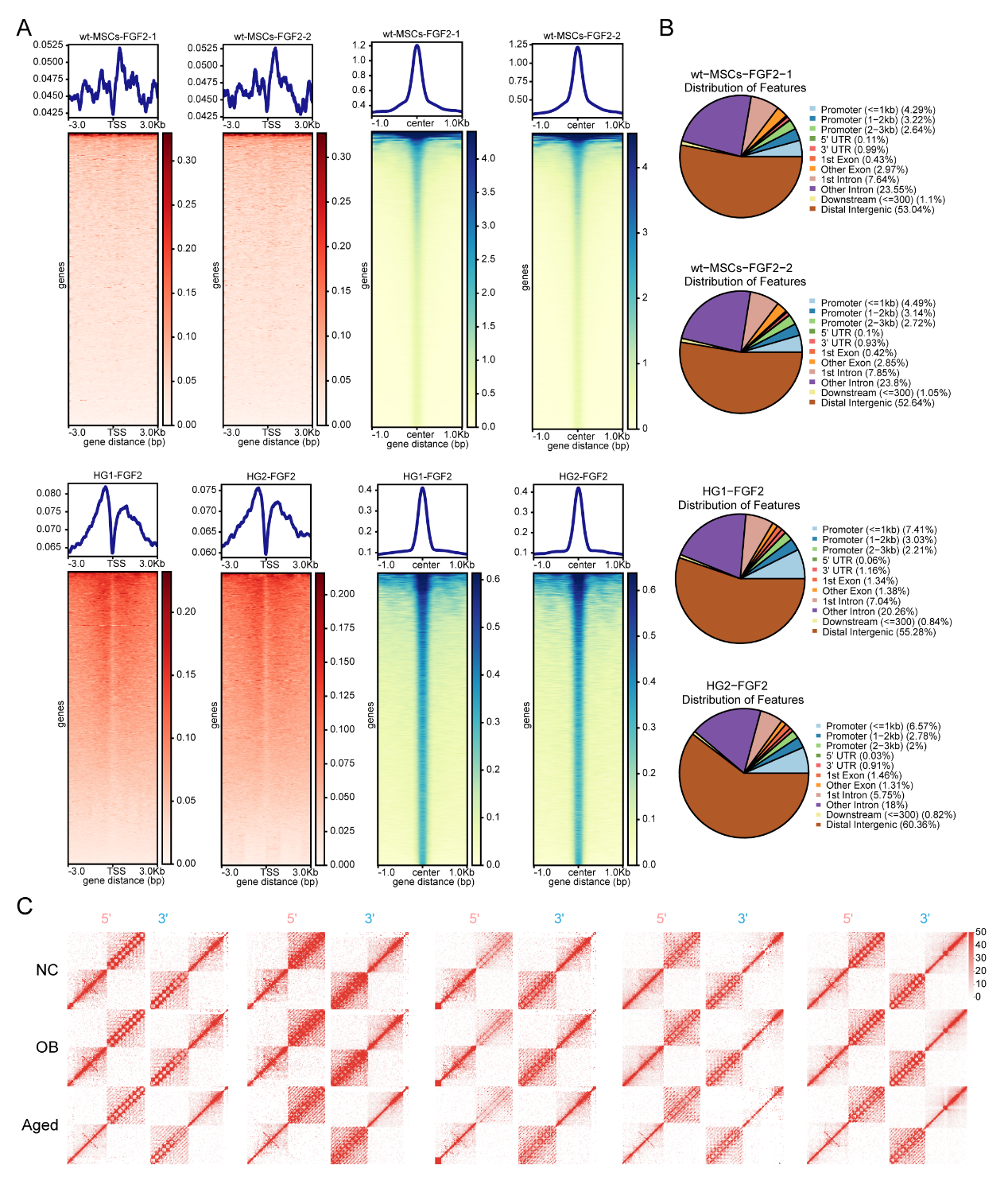


**Figure S6. phase separation FGF2 orchestrates rDNA region related chromatin architecture, related to Figure 6**

(A) Heatmaps and average profiles showing the distribution of FGF2 binding sites centered around transcription start sites (TSS) in wild-type MSCs (wt-MSCs) and 1,6 HD-treated MSCs (HG1 and HG2). (B) Pie charts depict the distribution of FGF2 binding sites across various genomic features in wild-type MSCs (wt-MSCs) and 1,6 HD-treated MSCs (HG1 and HG2). (C) Hi-C interaction matrices for 5’ and 3’ regions of ribosomal RNA gene loci are shown for NC, OB, and aged MSCs. The heatmaps illustrate the changes in chromatin interactions across these loci.


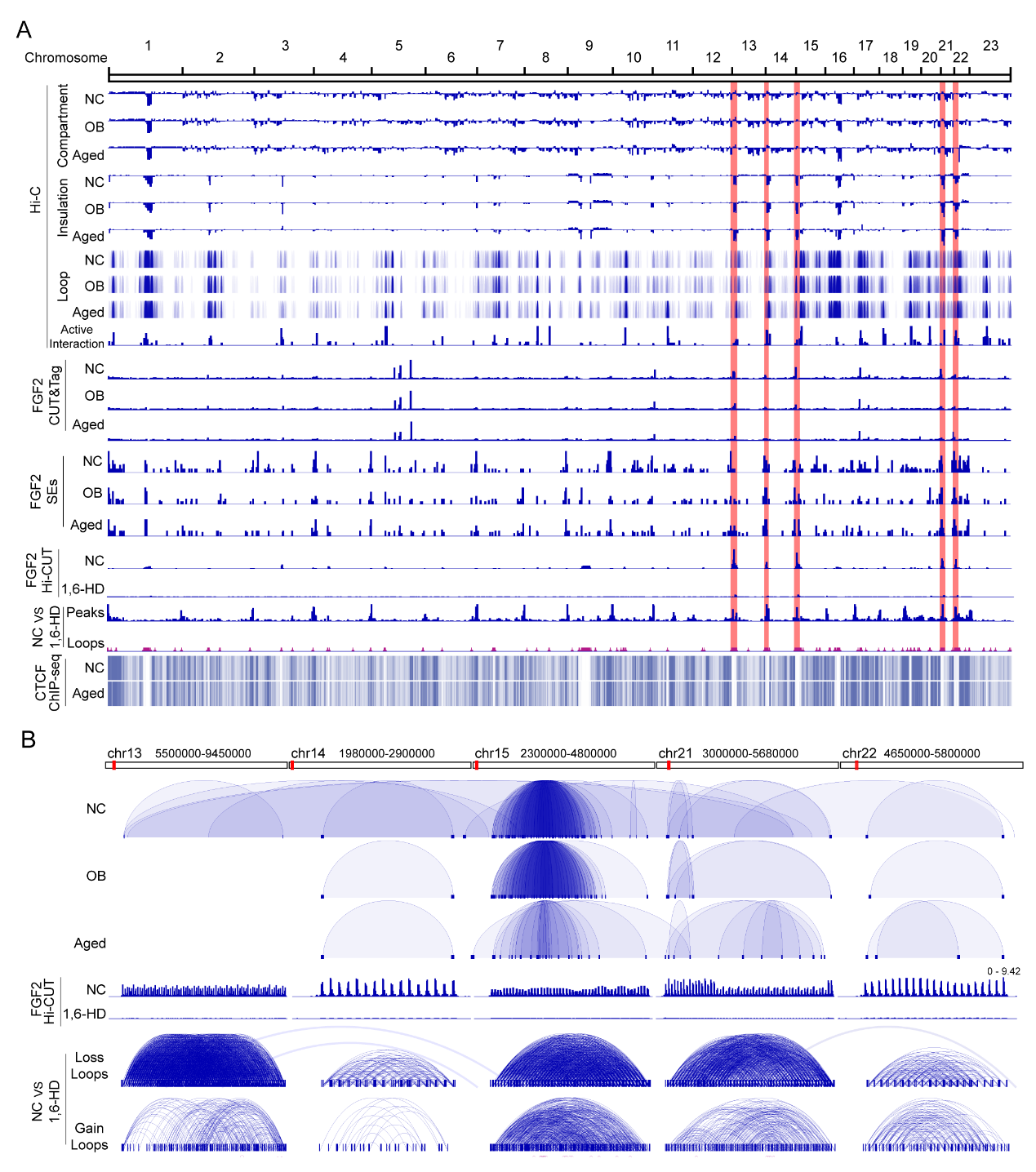
**Figure S7. Genome-wide analysis of chromatin architecture and FGF2 binding in MSCs fate conversion**

(A) The top panels show Hi-C compartment scores, insulation profiles, loop interactions, and active interaction regions across all chromosomes. The middle panels display FGF2 CUT&Tag peaks, super-enhancer (SE) regions, and FGF2 Hi-Cut interactions for NC, OB, and aged groups, highlighting significant binding and interaction differences. The bottom panels compare CTCF ChIP-seq peaks between NC and aged groups, showing changes in chromatin loops and CTCF occupancy. Red bars highlight specific chromosomal regions of interest, indicating alterations in chromatin structure and FGF2 activity associated with aging and osteoblast differentiation. (B) Chromatin looping dynamics across chromosomes 13, 14, 15, 21, and 22 in different MSCs states. The arc diagrams illustrate chromatin loop formations in NC, OB, and aged MSCs across selected chromosomal regions. The density and span of loops represent chromatin interactions. The bottom panels shown the FGF2 Hi-CUT signal for NC and 1,6-HD treated MSCs. Loop gain and loss events between NC and 1,6-HD-treated cells are also depicted, highlighting the dynamic reorganization of chromatin architecture in response to FGF2 and chemical perturbation.


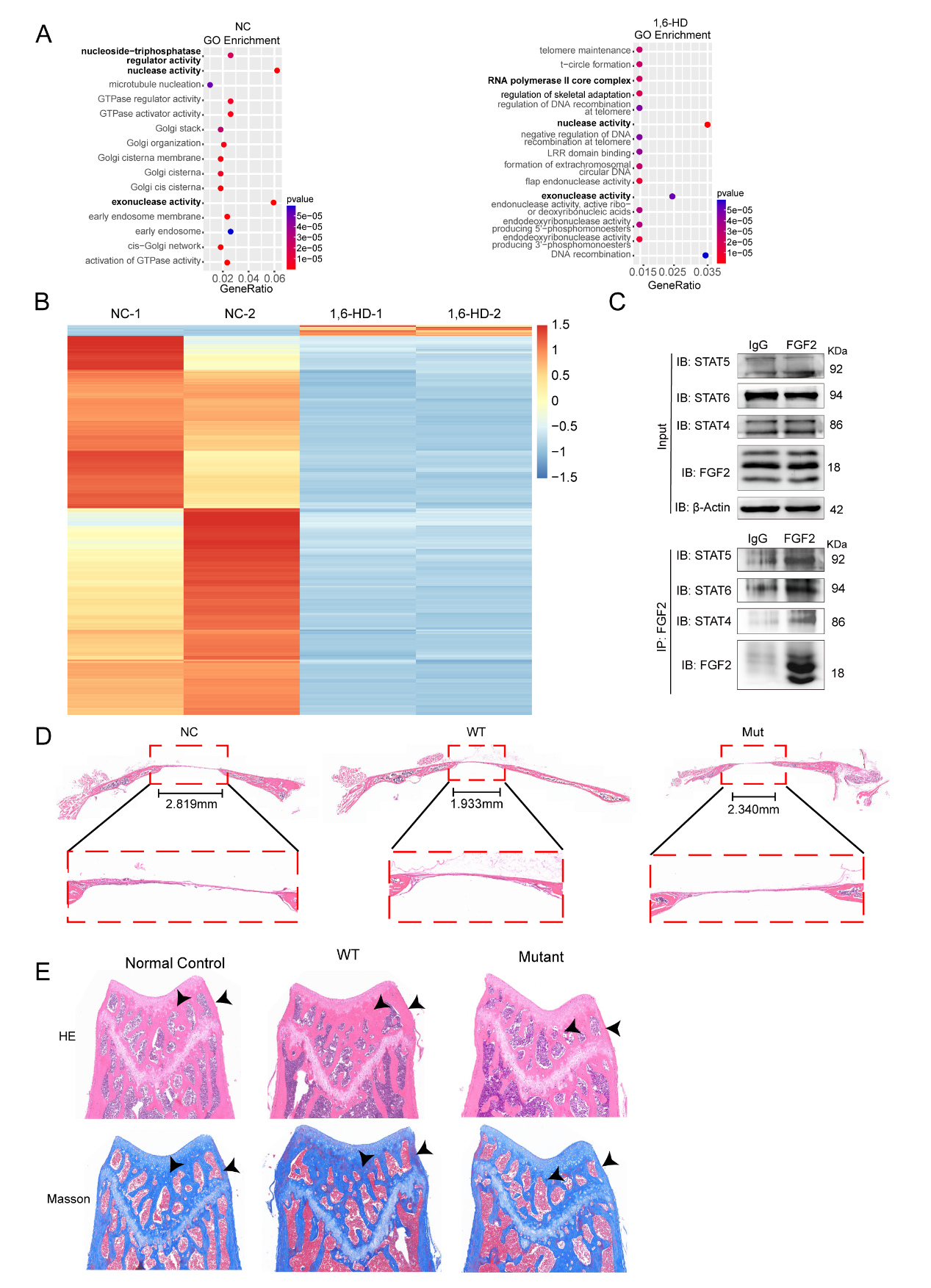
 **Figure S8. FGF2 activates rDNA dynamic in coordination with STAT5, related to Figure 7**

(A) Gene Ontology (GO) enrichment analysis of differentially expressed genes in NC and 1,6-HD-treated BMSCs. The dot plots display the top enriched GO terms related to molecular functions and biological processes. (B) Heatmap of differential gene expression in in NC and 1,6-HD treated MSCs across two biological replicates (NC-1, NC-2, 1,6-HD-1, 1,6-HD-2). (C) Immunoprecipitation of STAT4, STAT5, STAT6 using an anti-FGF2 antibody in normal MSCs. (D) Representative H&E-stained coronal sections of 3-mm cranial defects in aged mice at 4 weeks post-injection with PBS, wild-type FGF2, or mutant FGF2. (E) Representative H&E and Masson's trichrome staining of femoral germinal center tissues in aged mice at 4 weeks post-injection with PBS, wild-type FGF2, or mutant FGF2.
