# Supplementary material for: Nuclear FGF2 orchestrates phase separation-mediated rDNA chromatin architecture to control BMSCs cell fate
Source: Bone Res. 2025 Sep 24;13:80. doi: 10.1038/s41413-025-00451-y (PMC12460815; doi:10.1038/s41413-025-00451-y)
Supplement: Supplementary file 2 — Supplementary Tables [file 41413_2025_451_MOESM2_ESM.docx]

**Table S1. qPCR primers & Knockdown & Plasmid sequences**

| **qPCR primers** | |
| --- | --- |
| QRT-β-actin-F | GTGGCCGAGGACTTTGAT |
| QRT-β-actin-R | CCTGTAACAACGCATCTCAT |
| QRT-human45S-F | TGTCAGGCGTTCTCGTCTC |
| QRT-human45S-R | AGCACGACGTCACCACATC |
| QRT-human28S-F | AGAGGTAAACGGGTGGGGTC |
| QRT-human28S-R | GGGGTCGGGAGGAACGG |
| QRT-human18S-F | GATGGTAGTCGCCGTGCC |
| QRT-human18S-R | GCCTGCTGCCTTCCTTGG |
| QRT-human5.8S-F | GGCTCGTGCGTCGATGAAGAAC |
| QRT-human5.8S-R | AGTGCGTTCGAAGTGTCGATGATC |
| **Genes knockdown related target sequences** | |
| shFGF2-1 | GACCCTCACATCAAGCTACAA |
| shFGF2-2 | CTATCAAAGGAGTGTGTGCTA |
| shKPNB1-1 | GGAAGTGTTGGGTGGTGAATT |
| shKPNB1-2 | CAGTGTAGTTGTTCGAGAT |
| shSTAT5-1 | CCTGTGGAACCTGAAACCATT |
| shSTAT5-2 | GAGAAGTTCACAGTCCTGTTT |
| **rRNA-promoter/enhancer luciferase activity related dual-plasmid sequences** | |
| Dual-luciferase reporter plasmid-1: 45S promoter (PGL4.23) | CTCGGGAAGAGCTTCTCGACTCACGGTTTCGCTTTCGCGGTCCACGGGCCGCCCTGCCAGCCGGATCTGTCTCGCTGACGTCCGCGGCGGTTGTCGGGCTCCATCTGGCGGCCGCTTTGAGATCGTGCTCTCGGCTTCCGGAGCTGCGGTGGCAGCTGCCGAGGGAGGGGACCGTCCCCGCTGTGAGCTAGGCAGAGCTCCGGAAAGCCCGCGGTCGTCAGCCCGGCTGGCCCGGTGGCGCCAGAGCTGTGGCGCGTCGCTTGTGAGTCACAGCTCTGGCGTGCAGGTTTATGTGGGGGAGAGGCTGTCGCTGCGCTTCTGGGCCCGAGGCGGGCGTGGGGCTGCCCGGGCCGGTCGACCAGCGCGCCGTAGCTCCCGAGGCCCGAGCCGCGACCCGCGGGGACCCGCCGCGCGTGGCGCGGGAGGCTGGGGACGCCCTTCCCGGCCCGGTCGCGGGTCCGCGCTCATCCTGGCCGTCTGAGGCGGCGGCCGAATTCGTTTCCGAGTCCCCGTGGGGAGCCGGGGACCGTCCCGCCCCCGTCCCCCGGGTGCCGGGGAGCGGTCCCCGGGCCGGGCCGCGGTCCCTCTGCCGCGATCCTTTCTGGCGAGTCCCCGTGCGGAGTCGGAGAGCGCTCCCTGAGCGCGCGTGCGGCCCGAGAGGTCGCGCCTGGCCGGCCTTCGGTCCCTCGTGTGTCCCGGTCGTAGGAGGGGCCGGCCGAAAATGCTTCCGGCTCCCGCTCTGGAGACACGGGCCGGCCCCCTGCGTGTGGCACGGGCGGCCGGGAGGGCGTCCCCGGCCCGGCGCTGCTCCCGCGTGTGTCCTGGGGTTGACCAGAGGGCCCCGGGCGCTCCGTGTGTGGCTGCGATGGTGGCGTTTTTGGGGACAGGTGTCCGTGTCGCGCGTCGCCTGGGCCGGCGGCGTGGTCGGTGACGCGACCTCCCGGCCCCGGGGGAGGTATATCTTTCGCTCCGAGTCGGCATTTTGGGCCGCCGGGTTATT |
| Dual-luciferase reporter plasmid-2: chr13 SEs (PGL4.23) | TTGATATTTTATGTACACTATAGAATATATATTTGGTGTACTTTGATATTTTATGTACAGAATAAAATATATATTTGATGTACTTTCATATTTTATGTACAGCATATAATACATGCTTTGGGTACTTTGATATTTTTTGTACAGTATAGAATATATACCTTGATATTTTTTGTATAGTATAGAATATATACCTTGGGTACTTTGATATTTTATGTACAGTATATAATATATGGTTTGTGAACTTTGATATTTCATGTAGAGTATAAAATATATATTTGGGGTACATTGATATTTTATGTACAGTATATAATCTATATTTGATGTACTTTCATATTTTATGTACCTAATATATATTTGATGTAATTTCATATTTTATGTACAGTATATAATTTATAGTTTGTGTACTTTGTTATTTTATGTACACTATAGAATATATATCTGGTGTACTTTGATATTTTATGTACAGATATAATACATGCTTTGGGTACTTTGATATTTTTTGTACAGTATAGAATATATACCTTGGGTACATTGATATTTTATGTACAGTATATAATATATAGTTTGTGAACTTTGATATTTCATGTACAGTATATAATATATATTTGGGTACTTTGATATTTTATGTACAGTATATAATCTATATTTGATGTACTTTCATATTTTATGTACCTAATATAATATATATTTGATGTAATTTCATATTTTATGTACAGTATAATATTTGGTGTACTTTGATATTTTATGTACAGAATAAAATATATATTTGATGTACTTTCATATTTTATGGACAGTATATAATACATGCTTTGGGTACTTTGATATTTTTTGTACAGTATAGAATATATACCTTGGGTACTTTGATATTTTATGTGCAGTATATAATATATAGTTTGTGAACTTTGATATTTCATGTACAGTATAATATATATATTTGGGGTACTTTGGTATTTTATATACAGTATATAATCTATATTTGATGTACTTTCATATTTTCTGTACCTAATATAATATATATTTGATGTAATTTCATATTTTATGTACAGTATATAATGTATACTTTGGGTACTTTGATATTTTATGTACACTATAGAATATATATTTGCTGTACTTTGATATTATATGTACAGAATAAAATATGTATTTGATGTACTTTCATATTTTATGTACAGTATATAATACATGCTTTGGGTACTTTGATATTTTTTGTACAGTATAGAATATATACCTTGAGTACTTTGATATTTTATGTACAGTATATAATATATAGTTTGTGAACTATGATATTTCATGTACAGTATAAAATATATATTTGAGAATAAAATATATATTTCATGTAATTTCATATTTTATGTACAGTATATAATACATGCTTTGGGTACTTTGATATTTTTTATATACAGTATATAATACATGCTTTGGGTACTTTGATATTTTTTGTGCAGTATAGAATATATACCTTGGGTACTTTGATATTTTATGTACAGTATATAATATATAGTTTTGATATTTTTTGTACAGTATAGAATACATACCTTGGGTACTTTGATATTTTATGTACAGTATACAATATATGGTTTGTGAACTTTGATATTTCATGTAGAGTATAAAATATATATTTGGGGTACTTTGATATTTTATGTACAGTATATATTTGATGTACTTTCATATTTTAGGTACAGTATATAATACATGCTTTGGGTACTTTGATATTTTTTGTACAGTATAGAATGTATACCTTGGGTACTTTGATATTTTATGTACAGTATATAATATATAGTTTGTGAACTTTGATATTTCATGTACAGTATAAAATATATATTTGGGGTACTTTGGTATTTAATGTACAGTATATAATCT |
| Dual-luciferase reporter plasmid-3: chr15 SEs (PGL4.23) | AGTATTTTATTGAATAAGATTTGCTCACAGAAAAATAAGTTTAAATCTACAATGAATGCCAGACCCTACAGCAGAAAGCAATTTTCTCACTTTTCCACACACAATGGTTCCTACTAAGTGAAAAAAAAGCCATAAAATTTCATTCACAAATGTACTACTCTGTCTCAAAACATCTCACGTAATCATGCACTGTACTAAAGCCATCAGATCAGTTCTTCAGTCAGGTTAAAGAAGTATCCCTCTAATAACTGACTCTTATAATGCTATCAATATGCACTCCCAATCAGTCTGCCATTGTTAATGGTGTACAGCATTACTGAATACAACGGAATTGATGACGCCCATATCCACAGACAAACCGTGACTTATGATGGTTTGATTTATATGAATCTTGAGGAGTCAGGGCTGGGGGACCGCTCATATTCTCCCGAGACCTGTGAGTCTCTGGTGGACTCCTGGGTGCATGGGGCTGGCTCCCGCAGGAACCTGGGGATGGCTGGAGAGTAACTGGGAGCCACAGGAGGGTCCCTGAGGCCTGGGGGTGAAGAGATGCAAGACACAGGGGTGGAGCAGCGTGAGGCTCGTGAGTTTGTAGGTGATTCCTGGGTGTGGGGGGCTGACTCCAGCTGAAATCTGGGGTTGTTTGGAGAGTAGCTGGGAGACACAGGAGACCCCCCGAGAGCTGGGGGTGAGCTGCTGGGTGATGGCAGTAAGAACATGTGGTATATTATTGATGAACGTGGGGACTCTGAGGAGTCCTCAGAGGAGGACACGGCAGAGCCCAATGGCTTCATTGATTGCCCATCACGGTGAGGACAGGGAAATGGGAGCTTGTGGGATTCTGGTGATGACAGAGGTGAGTGTGGTGAAGCCCTAGGGGATGGTGAATGGTAGTTCCGGATCCCTGGTGAGGAGCTTCCCCTTAAGCCTGAGTTTCTGAGAGGGGAGAGGGAGAAGCTGGGTGAGGCTCGCATGGACCTTGGGGTGTCCGTGCTGGGGGACCGTTCATAAGAAGAGCCAGACAAGACCCTACTGTTCTTAGGTGCAGACATGATTAGGAAACCTGCAGCTCCCAGGGGCCCCTACTAATGTTCTAACTCGCAGAAGGAAGGAGTGTGTGTGCGTGTGTGCGTGTGCGTGTGTGTGTGTGTGTGTGTGTGCTTGTGTGTGTGCGGTGTGAGGTATGTGCCCCTTAAGAAAATGGAAATCAAACAACCAATGGGACAGAGAGACAGACAGACAGACAGAGATTCACTTGCCCAAGTGTTCTGTCCTGTCCTCTGAATCCGCTTCCAAGACGCAAGACGCTGTGAGCTCCAAGTCCACGCAGAGTCCGCCAAACGCTGCGGCCGCTGATCCGCTCCGCGAAGATCTGAGTACAGGCCAGCCAGGGTGGGTTTAAATAGCCTCGGGCGCAGCCTAGCAGCGGAAAGGGCGGAGCTTCACTCCTCCTTTCCATCAGTCACCCCCAAGGGAGCTTGGGGGATTCTGGTGATGACAGAGGTGAGTGTGGTGAAGCCCTAGGGGATGGTGAATGGTAGTTCCGGATCCCTGGTGAGGAGCTTCCCCTTAAGCCTGAGTTTCTGAGAGGGGAGAGGGAGAAGCTGGGTGAGGCTCGCATGGACCTTGGGGTGTCCGTGCTGGGGGACCGTTCATAAGAAGAGCCAGACAAGACCCTACTGTTCTTAGGTGCAGACATGATTAGGAAACCTGCAGCTCCCAGGGGCCCCTACTAATGTTCTAACTCGAAGAAGGAAGGAGTGTGTGTGCGTGTGTGCGTGTGCGTGTGTGTGTGTGTGTGCTTGTGTGTGTGCGGTGTGAGGTATGTGCCCCTTAAGAAAATGGAAATCAACCAACCAATGAGACAGAGAGACAGACAGACAGACAGAGATTCACTTGCCCAAGTGTTCTGTCCTGTCCTCTGAATCCGCTTCCAAGTCGCAAGACGCTGTGAGCTCCAAGTCCACGCAGAGTCCGCCAAACGCTGCGGCCGCTGATCCGCTCCGCGAAGATCTGAGTACAGGCCAGCCAGGGTGGGTTTAGATAGCCTCGGGCGCAGCCTAGCAGCGGAAAGGGCGGAGCTTCACTCCTCCTTTCCATCAGTCACCCCCAACTTTCCCAGGCTACACCTCGTAGGAAACTGTTCTCCTGCTTTGATTTCATGCGCCCGTTCATAAGAAGAGCCAGACAAGACCCTACTGTTCTTAGGTGCAGACATGATTAGGAAACCTGCAGCTCCCAGGGGCCCCCACTAATGTTCTAACTCGAAGAAGGAAGGAGTGTGTGTGCGTGTGTGCGTGTGCGTGTGTGTGTGTGTGTGTGTGTTTGTGTGTGTGCGGTGTGAGGTATGGGCCCCTTAAGGAAATGGAAATCAACCAACCAATGGGACAGACAGACAGACAGACAGACAGAGATTCACTTGCCCAAGTGTTCTGTCCTGTCCTCTGAATCCGCTTTCAAGTCGCAAGACGCTGTGAGCTCCAAGTCCACGCAGAGTCCGCCAAACGCTGCGGCCGCTGATCCGCTCCGCGAAGATCTGAGTACAGGCCAGCCAGGGTGGGTTTAAATAGCCTCGGGCGCAGCCTAGCAGCGGAAAGGGCGGAGCTTCACTCCTCCTTTCCATCAGTCACCCCCAACTTTCCCAGGCTACACCTCGTAGGAAACTGTTCTCCTGCTTTGATTTCATGCGCCACCTTTGGGACAATCTAAGAACTTACAGGTTTTCTTGGCCAGATTTATTAGGAATTGTATGCACTGAAA |
| Dual-luciferase reporter plasmid-4: 18S (PGL4.23) | TACCTGGTTGATCCTGCCAGTAGCATATGCTTGTCTCAAAGATTAAGCCATGCATGTCTAAGTACGCACGGCCGGTACAGTGAAACTGCGAATGGCTCATTAAATCAGTTATGGTTCCTTTGGTCGCTCGCTCCTCTCCTACTTGGATAACTGTGGTAATTCTAGAGCTAATACATGCCGACGGGCGCTGACCCCCTTCGCGGGGGGGATGCGTGCATTTATCAGATCAAAACCAACCCGGTCAGCCCCTCTCCGGCCCCGGCCGGGGGGCGGGCGCCGGCGGCTTTGGTGACTCTAGATAACCTCGGGCCGATCGCACGCCCCCCGTGGCGGCGACGACCCATTCGAACGTCTGCCCTATCAACTTTCGATGGTAGTCGCCGTGCCTACCATGGTGACCACGGGTGACGGGGAATCAGGGTTCGATTCCGGAGAGGGAGCCTGAGAAACGGCTACCACATCCAAGGAAGGCAGCAGGCGCGCAAATTACCCACTCCCGACCCGGGGAGGTAGTGACGAAAAATAACAATACAGGACTCTTTCGAGGCCCTGTAATTGGAATGAGTCCACTTTAAATCCTTTAACGAGGATCCATTGGAGGGCAAGTCTGGTGCCAGCAGCCGCGGTAATTCCAGCTCCAATAGCGTATATTAAAGTTGCTGCAGTTAAAAAGCTCGTAGTTGGATCTTGGGAGCGGGCGGGCGGTCCGCCGCGAGGCGAGCCACCGCCCGTCCCCGCCCCTTGCCTCTCGGCGCCCCCTCGATGCTCTTAGCTGAGTGTCCCGCGGGGCCCGAAGCgtttactttgaaaaaattagaGTGTTCAAAGCAGGCCCGAGCCGCCTGGATACCGCAGCTAGGAATAATGGAATAGGACCGCGGTTCTATTTTGTTGGTTTTCGGAACTGAGGCCATGATTAAGAGGGACGGCCGGGGGCATTCGTATTGCGCCGCTAGAGGTGAAATTCTTGGACCGGCGCAAGACGGACCAGAGCGAAAGCATTTGCCAAGAATGTTTTCATTAATCAAGAACGAAAGTCGGAGGTTCGAAGACGATCAGATACCGTCGTAGTTCCGACCATAAACGATGCCGACCGGCGATGCGGCGGCGTTATTCCCATGACCCGCCGGGCAGCTTCCGGGAAACCAAAGTCTTTGGGTTCCGGGGGGAGTATGGTTGCAAAGCTGAAACTTAAAGGAATTGACGGAAGGGCACCACCAGGAGTGGAGCCTGCGGCTTAATTTGACTCAACACGGGAAACCTCACCCGGCCCGGACACGGACAGGATTGACAGATTGATAGCTCTTTCTCGATTCCgtgggtggtggtgcatggcCGTTCTTAGTTGGTGGAGCGATTTGTCTGGTTAATTCCGATAACGAACGAGACTCTGGCATGCTAACTAGTTACGCGACCCCCGAGCGGTCGGCGTCCCCCAACTTCTTAGAGGGACAAGTGGCGTTCAGCCACCCGAGATTGAGCAATAACAGGTCTGTGATGCCCTTAGATGTCCGGGGCTGCACGCGCGCTACACTGACTGGCTCAGCGTGTGCCTACCCTACGCCGGCAGGCGCGGGTAACCCGTTGAACCCCATTCGTGATGGGGATCGGGGATTGCAATTATTCCCCATGAACGAGGAATTCCCAGTAAGTGCGGGTCATAAGCTTGCGTTGATTAAGTCCCTGCCCTTTGTACACACCGCCCGTCGCTACTACCGATTGGATGGTTTAGTGAGGCCCTCGGATCGGCCCCGCCGGGGTCGGCCCACGGCCCTGGCGGAGCGCTGAGAAGACGGTCGAACTTGACTATCTAGAGGAAGTAAAAGTCGTAACAAGGTTTCCGTAGGTGAACCTGCGGAAGGATCATTA |
| Dual-luciferase reporter plasmid-5: 5.8S (PGL4.23) | GACTCTTAGCGGTGGATCACTCGGCTCGTGCGTCGATGAAGAACGCAGCTAGCTGCGAGAATTAATGTGAATTGCAGGACACATTGATCATCGACACTTCGAACGCACTTGCGGCCCCGGGTTCCTCCCGGGGCTACGCCTGTCTGAGCGTCG |
| Dual-luciferase reporter plasmid-6: 28S (PGL4.23) | CGACCTCAGATCAGACGTGGCGACCCGCTGAATTTAAGCATATTAGTCAGCGGAGGAAAAGAAACTAACCAGGATTCCCTCAGTAACGGCGAGTGAACAGGGAAGAGCCCAGCGCCGAATCCCCGCCCCGCGGCGGGGCGCGGGACATGTGGCGTACGGAAGACCCGCTCCCCGGCGCCGCTCGTGGGGGGCCCAAGTCCTTCTGATCGAGGCCCAGCCCGTGGACGGTGTGAGGCCGGTAGCGGCCCCCGGCGCGCCGGGCCCGGGTCTTCCCGGAGTCGGGTTGCTTGGGAATGCAGCCCAAAGCGGGTGGTAAACTCCATCTAAGGCTAAATACCGGCACGAGACCGATAGTCAACAAGTACCGTAAGGGAAAGTTGAAAAGAACTTTGAAGAGAGAGTTCAAGAGGGCGTGAAACCGTTAAGAGGTAAACGGGTGGGGTCCGCGCAGTCCGCCCGGAGGATTCAACCCGGCGGCGGGTCCGGCCGTGTCGGCGGCCCGGCGGATCTTTCCCGCCCCCCGTTCCTCCCGACCCCTCCACCCGCCCTCCCTTCCCCCGCCGCCCCTCCTCCTCCTCCCCGGAGAAGGTGAAGGCCGGCGCGCTCGCCGGCCGAGGTGGGATCCCGAGGCCTCTCCAGTCCGCCGAGGGCGCACCACCGGCCCGTCTCGCCCGCCGCGCCGGGGAGGTGGAGCACGAGCGCACGTGTTAGGACCCGAAAGATGGTGAACTATGCCTGGGCAGGGCGAAGCCAGAGGAAACTCTGGTGGAGGTCCGTAGCGGTCCTGACGTGCAAATCGGTCGTCCGACCTGGGTATAGGGGCGAAAGACTAATCGAACCATCTAGTAGCTGGTTCCCTCCGAAGTTTCCCTCAGGATAGCTGGCGCTCTCGCAGACCCGACGCACCCCCGCCACGCAGTTTTATCCGGTAAAGCGAATGATTAGAGGTCTTGGGGCCGAAACGATCTCAACCTATTCTCAAACTTTAAATGGGTAAGAAGCCCGGCTCGCTGGCGTGGAGCCGGGCGTGGAATGCGAGTGCCTAGTGGGCCACTTTTGGTAAGCAGAACTGGCGCTGCGGGATGAACCGAACGCCGGGTTAAGGCGCCCGATGCCGACGCTCATCAGACCCCAGAAAAGGTGTTGGTTGATATAGACAGCAGGACGGTGGCCATGGAAGTCGGAATCCGCTAAGGAGTGTGTAACAACTCACCTGCCGAATCAACTAGCCCTGAAAATGGATGGCGCTGGAGCGTCGGGCCCATACCCGGCCGTCGCCGGCAGTCGAGAGTGGACGGGAGCGGCGGGGGCGGCGCGCGCGCGGGCCGCTGCGGTGAGCCTTGAAGCCTAGGGCGCGGGCCCGGGTGGAGCCGCCGCAGGTGCAGATCTTGGTGGTAGTAGCAAATATTCAAACGAGAACTTTGAAGGCCGAAGTGGAGAAGGGTTCCATGTGAACAGCAGTTGAACATGGGTCAGTCGGTCCTGAGAGATGGGCGAGCGCCGTTCCGAAGGGACGGGCGATGGCCTCCGTTGCCCTCGGCCGATCGAAAGGGAGTCGGGTTCAGATCCCCGAATCCGGAGTGGCGGAGATGGGCGCCGCGAGGCGTCCAGTGCGGTAACGCGACCGATCCCGGAGAAGCCGGCGGGAGCCCCGGGGAGAGTTCTCTTTTCTTTGTGAAGGGCAGGGCGCCCTGGAATGGGTTCGCCCCGAGAGAGGGGCCCGTGCCTTGGAAAGCGTCGCGGTTCCGGCGGCGTCCGGTGAGCTCTCGCTGGCCCTTGAAAATCCGGGGGAGAGGGTGTAAATCTCGCGCCGGGCCGTACCCATATCCGCAGCAGGTCTCCAAGGTGAACAGCCTCTGGCATGTTGGAACAATGTAGGTAAGGGAAGTCGGCAAGCCGTGAAGAAATTCAATGAAGCGCGGGTAAACGGCGGGAGTAACTATGACTCTCTTAAGGTAGCCAAATGCCTCGTCATCTAATTAGTGACGCGCATGAATGGATGAACGAGATTCCCACTGTCCCTACCTACTATCCAGCGAAACCACAGCCAAGGGAACGGGCTTGGCGGAATCAGCGGGGAAAGAAGACCCTGTTGAGCTTGACTCTAGTCTGGCACGGTGAAGAGACATGAGAGGTGTAGAATAAGTGGGAGGCCCCCGGCGCCCCCCCGGTGTCCCCGCGAGGGGCCCGGGGCGGGGTCCGCCGGCCCTGCGGGCCGCCGGTGAAATACCACTACTCTGATCGTTTTTTCACTGACCCGGTGAGGCGGGGGGGCGAGCCCCGAGGGGCTCTCGCTTCTGGCGCCAAGCGCCCGGCCGCGCGCCGGCCGGGCGCGACCCGCTCCGGGGACAGTGCCAGGTGGGGAGTTTGACTGGGGCGGTACACCTGTCAAACGGTAACGCAGGTGTCCTAAGGCGAGCTCAGGGAGGACAGAAACCTCCCGTGGAGCAGAAGGGCAAAAGCTCGCTTGATCTTGATTTTCAGTACGAATACAGACCGTGAAAGCGGGGCCTCACGATCCTTCTGACCTTTTGGGTTTTAAGCAGGAGGTGTCAGAAAAGTTACCACAGGGATAACTGGCTTGTGGCGGCCAAGCGTTCATAGCGACGTCGCTTTTTGATCCTTCGATGTCGGCTCTTCCTATCATTGTGAAGCAGAATTCACCAAGCGTTGGATTGTTCACCCACTAATAGGGAACGTGAGCTGGGTTTAGACCGTCGTGAGACAGGTTAGTTTTACCCTACTGATGATGTGTTGTTGCCATGGTAATCCTGCTCAGTACGAGAGGAACCGCAGGTTCAGACATTT |
| **plasmid and related sequence** | |
| pCDH-FGF2 | Kozak sequence + ATGGCAGCCGGGAGCATCACCACGCTGCCCGCCTTGCCCGAGGATGGCGGCAGCGGCGCCTTCCCGCCCGGCCACTTCAAGGACCCCAAGCGGCTGTACTGCAAAAACGGGGGCTTCTTCCTGCGCATCCACCCCGACGGCCGAGTTGACGGGGTCCGGGAGAAGAGCGACCCTCACATCAAGCTACAACTTCAAGCAGAAGAGAGAGGAGTTGTGTCTATCAAAGGAGTGTGTGCTAACCGTTACCTGGCTATGAAGGAAGATGGAAGATTACTGGCTTCTAAATGTGTTACGGATGAGTGTTTCTTTTTTGAACGATTGGAATCTAATAACTACAATACTTACCGGTCAAGGAAATACACCAGTTGGTATGTGGCACTGAAACGAACTGGGCAGTATAAACTTGGATCCAAAACAGGACCTGGGCAGAAAGCTATACTTTTTCTTCCAATGTCTGCTAAGAGCTGA + termination codon |
| pEZ-M98-STAT5 | Kozak sequence + ATGGCGGGCTGGATCCAGGCCCAGCAGCTGCAGGGAGACGCGCTGCGCCAGATGCAGGTGCTGTACGGCCAGCACTTCCCCATCGAGGTCCGGCACTACTTGGCCCAGTGGATTGAGAGCCAGCCATGGGATGCCATTGACTTGGACAATCCCCAGGACAGAGCCCAAGCCACCCAGCTCCTGGAGGGCCTGGTGCAGGAGCTGCAGAAGAAGGCGGAGCACCAGGTGGGGGAAGATGGGTTTTTACTGAAGATCAAGCTGGGGCACTACGCCACGCAGCTCCAGAAAACATATGACCGCTGCCCCCTGGAGCTGGTCCGCTGCATCCGGCACATTCTGTACAATGAACAGAGGCTGGTCCGAGAAGCCAACAATTGCAGCTCTCCGGCTGGGATCCTGGTTGACGCCATGTCCCAGAAGCACCTTCAGATCAACCAGACATTTGAGGAGCTGCGACTGGTCACGCAGGACACAGAGAATGAGCTGAAGAAACTGCAGCAGACTCAGGAGTACTTCATCATCCAGTACCAGGAGAGCCTGAGGATCCAAGCTCAGTTTGCCCAGCTGGCCCAGCTGAGCCCCCAGGAGCGTCTGAGCCGGGAGACGGCCCTCCAGCAGAAGCAGGTGTCTCTGGAGGCCTGGTTGCAGCGTGAGGCACAGACACTGCAGCAGTACCGCGTGGAGCTGGCCGAGAAGCACCAGAAGACCCTGCAGCTGCTGCGGAAGCAGCAGACCATCATCCTGGATGACGAGCTGATCCAGTGGAAGCGGCGGCAGCAGCTGGCCGGGAACGGCGGGCCCCCCGAGGGCAGCCTGGACGTGCTACAGTCCTGGTGTGAGAAGTTGGCCGAGATCATCTGGCAGAACCGGCAGCAGATCCGCAGGGCTGAGCACCTCTGCCAGCAGCTGCCCATCCCCGGCCCAGTGGAGGAGATGCTGGCCGAGGTCAACGCCACCATCACGGACATTATCTCAGCCCTGGTGACCAGCACATTCATCATTGAGAAGCAGCCTCCTCAGGTCCTGAAGACCCAGACCAAGTTTGCAGCCACCGTACGCCTGCTGGTGGGCGGGAAGCTGAACGTGCACATGAATCCCCCCCAGGTGAAGGCCACCATCATCAGTGAGCAGCAGGCCAAGTCTCTGCTTAAAAATGAGAACACCCGCAACGAGTGCAGTGGTGAGATCCTGAACAACTGCTGCGTGATGGAGTACCACCAAGCCACGGGCACCCTCAGTGCCCACTTCAGGAACATGTCACTGAAGAGGATCAAGCGTGCTGACCGGCGGGGTGCAGAGTCCGTGACAGAGGAGAAGTTCACAGTCCTGTTTGAGTCTCAGTTCAGTGTTGGCAGCAATGAGCTTGTGTTCCAGGTGAAGACTCTGTCCCTACCTGTGGTTGTCATCGTCCACGGCAGCCAGGACCACAATGCCACGGCTACTGTGCTGTGGGACAATGCCTTTGCTGAGCCGGGCAGGGTGCCATTTGCCGTGCCTGACAAAGTGCTGTGGCCGCAGCTGTGTGAGGCGCTCAACATGAAATTCAAGGCCGAAGTGCAGAGCAACCGGGGCCTGACCAAGGAGAACCTCGTGTTCCTGGCGCAGAAACTGTTCAACAACAGCAGCAGCCACCTGGAGGACTACAGTGGCCTGTCCGTGTCCTGGTCCCAGTTCAACAGGGAGAACTTGCCGGGCTGGAACTACACCTTCTGGCAGTGGTTTGACGGGGTGATGGAGGTGTTGAAGAAGCACCACAAGCCCCACTGGAATGATGGGGCCATCCTAGGTTTTGTGAATAAGCAACAGGCCCACGACCTGCTCATCAACAAGCCCGACGGGACCTTCTTGTTGCGCTTTAGTGACTCAGAAATCGGGGGCATCACCATCGCCTGGAAGTTTGACTCCCCGGAACGCAACCTGTGGAACCTGAAACCATTCACCACGCGGGATTTCTCCATCAGGTCCCTGGCTGACCGGCTGGGGGACCTGAGCTATCTCATCTATGTGTTTCCTGACCGCCCCAAGGATGAGGTCTTCTCCAAGTACTACACTCCTGTGCTGGCTAAAGCTGTTGATGGATATGTGAAACCACAGATCAAGCAAGTGGTCCCTGAGTTTGTGAATGCATCTGCAGATGCTGGGGGCAGCAGCGCCACGTACATGGACCAGGCCCCCTCCCCAGCTGTGTGCCCCCAGGCTCCCTATAACATGTACCCACAGAACCCTGACCATGTACTCGATCAGGATGGAGAATTCGACCTGGATGAGACCATGGATGTGGCCAGGCACGTGGAGGAACTCTTACGCCGACCAATGGACAGTCTTGACTCCCGCCTCTCGCCCCCTGCCGGTCTTTTCACCTCTGCCAGAGGCTCCCTCTCATGA + termination codon |

**Table S2. Key Resources Table**

| **Reagent or Resources** | **Source** | **Identifier** |
| --- | --- | --- |
| **Antibodies** | | |
| Anti-FGF2 | Abcam | #ab208687; RRID: AB_3099488 |
| Anti-KPNB1 | Abcam | #ab2811; RRID: AB_2133989 |
| Anti-P53 | proteintech | #10442-1-AP; RRID: AB_2206609 |
| Anti-P21 | proteintech | #10355-1-AP; RRID: AB_2077682 |
| Anti-P16 | proteintech | #10883-1-AP; RRID: AB_2078303 |
| β-actin | proteintech | #66009-1-Ig; RRID: AB_2687938 |
| Cyclin E | Santa Cruz Biotechnology | #sc-377100; RRID: AB_2923122 |
| Cyclin D | Santa Cruz Biotechnology | #sc-8396; RRID: AB_627344 |
| p-Tyr | Santa Cruz Biotechnology | #sc-7020; RRID: AB_628123 |
| p-Ser | Santa Cruz Biotechnology | #sc-81514; RRID: AB_1128624 |
| STAT4 | proteintech | #13028-1-AP; RRID: AB_2196604 |
| STAT5 | proteintech | #13179-1-AP; RRID: AB_2196760 |
| STAT6 | proteintech | #51073-1-AP; RRID: AB_2197244 |
| MED1 | Affinity Biosciences | #DF6578; RRID: AB_2838540 |
| H3K27ac | Abcam | #ab4729; RRID: AB_2118291 |
| IgG (IP) | proteintech | #30000-0-AP; RRID: AB_2819035 |
| Cy3–conjugated Affinipure Goat Anti-Rabbit IgG(H+L) | proteintech | #SA00009-2; RRID: AB_2890957 |
| Fluorescein (FITC)–conjugated Affinipure Goat Anti-Mouse IgG(H+L) | proteintech | # SA00003-1; RRID: AB_2890896 |
| Multi-rAb HRP-Goat Anti-Rabbit Recombinant Secondary Antibody (H+L) | proteintech | #RGAR001; RRID: AB_3073505 |
| Multi-rAb HRP-Goat Anti-Mouse Recombinant Secondary Antibody (H+L) | proteintech | #RGAM001; RRID: AB_3068333 |
| **Chemicals, peptides, and recombinant proteins** | | |
| Gibco™ BASIC MEM α | Thermo Fisher | #C12571500BT |
| DMEM (High Glucose) | Solarbio | #11995 |
| PBS | Solarbio | #P1020 |
| 0.25% Trypsin-EDTA | Beyotime | #C0203 |
| Fetal Bovine Serum | ScienCell | #0500 |
| Fetal Bovine Serum (Prime) | ExCell Bio | #FSP500 |
| Penicillin-streptomycin | Beyotime | #C0222 |
| Lipofectamine 2000 | Thermo Fisher | #11668-019 |
| RNAiso Plus | Takara | #9109 |
| Antifade Mounting Medium | LEAGENE | # IH0252 |
| DAPI | Beyotime | #C1002 |
| Hoechst 33342 | Beyotime | #C1029 |
| Rhodamine B | Solarbio | #R8040 |
| SDS-PAGE Protein Sample Loading Buffer | Beyotime | # P0288 |
| Coomassie Blue Fast Staining Solution | Beyotime | # P0017 |
| PEG8000 | Beyotime | #P8260 |
| Non-denatured Gel Protein Sample Loading Buffer | Beyotime | #P0292 |
| AceQ qPCR SYBR green Master Mix | Vazyme | #Q111-02 |
| 2×Taq Master Mix | Vazyme | #P112-01 |
| HiScript II QRT SuperMix for qPCR | Vazyme | #R222 |
| Polybrene | Beyotime | #ST1380 |
| Puromycin | Beyotime | #ST551 |
| Protease and phosphatase inhibitor cocktail | Beyotime | #P1045 |
| Protein A/G PLUS-Agarose | Santa Cruz Biotechnology | #sc-2003 |
| Bovine Serum Albumin Ⅴ | Solarbio | #A8020 |
| RIPA Lysis Buffer | Beyotime | #P0013B |
| Gelatin Aqueous Solution | Solarbio | #G0040 |
| Cell Counting Kit-8 (CCK-8) | TargetMol | #C0005 |
| Human FGF2 Recombinant protein | DGpeptides | #H20221020379 |
| **Critical commercial assays** | | |
| Hyperactive Universal CUT&Tag Assay Kit for Illumina Pro | Vazyme | #TD904 |
| TruePrep Index Kit V2 | Vazyme | #TD202 |
| Luc-Pair™ Duo-Luciferase HS Assay Kit | GeneCopoeia | #LF004 |
| **Deposited data** |  |  |
| Cut&Tag, Hi-C, and Hi-Cut data | This paper | SRA: PRJCA029726 |
| Mass Spectrometric | This paper | iProX: IPX0009728000 |
| Single cell RNA seq | This paper | GSM3439738 and GSM3439737 |
| ChIP-seq (H3K27ac and MED1) | This paper | GSE113253 |
| ChIP-seq (STAT5) | This paper | GSE97938 |
| ChIP-seq (CTCF) | This paper | GSE144195 |
| **Experimental models: Cell lines** | | |
| Human alveolar bone derived MSCs | This paper | available upon request |
| Mouse bone marrow derived MSCs | This paper | available upon request |
| HEK293T | Cas9X | #TCH-C101 |
| **Experimental models: Organisms/strains** | | |
| C57BL/6JGpt | GemPharmatech | # N000013 |
| **Oligonucleotides** | | |
| sh-RNA sequences | See Table S1 | N/A |
| Primers used in RT-qPCR  analysis | See Table S1 | N/A |
| **Recombinant DNA** | | |
| pCDH-CMV | Addgene | #72265; RRID: Addgene_72265 |
| pLKO.1-puro | Addgene | #8453; RRID: Addgene_8453 |
| psPAX2 | Addgene | #12260; RRID: Addgene_12260 |
| pMD2.G | Addgene | #12259; RRID: Addgene_12259 |
| pVSV-G | Addgene | #138479; RRID: Addgene_138479 |
| pRSV-Rev | Addgene | #12253; RRID: Addgene_12253 |
| gag/pol | Addgene | #14887; RRID: Addgene_14887 |
| pCDH-FGF2 | This paper | N/A |
| pLKO.1-sh-FGF2-1 | This paper | N/A |
| pLKO.1-sh-FGF2-2 | This paper | N/A |
| pLKO.1-sh-KPNB1-1 | This paper | N/A |
| pLKO.1-sh-KPNB1-2 | This paper | N/A |
| PGL4.23[luc2/minP] Vector | GenScript | #PO: C3177LCRG0 |
| Dual-luciferase reporter plasmid-1: 45S promoter (PGL4.23) | GenScript | #PO: C3177LCRG0 |
| Dual-luciferase reporter plasmid-2: chr13 SEs (PGL4.23) | GenScript | #PO: C3177LCRG0 |
| Dual-luciferase reporter plasmid-3: chr15 SEs (PGL4.23) | GenScript | #PO: C3177LCRG0 |
| Dual-luciferase reporter plasmid-4: 18S (PGL4.23) | GenScript | #PO: C3177LCRG0 |
| Dual-luciferase reporter plasmid-5: 5.8S (PGL4.23) | GenScript | #PO: C3177LCRG0 |
| Dual-luciferase reporter plasmid-6: 28S (PGL4.23) | GenScript | #PO: C3177LCRG0 |
| GV298-congtrol | Genechem | #PO: GIEE0438146 |
| GV298-sh-STAT5-1 | Genechem | #PO: GIEE0438146 |
| GV298-sh-STAT5-2 | Genechem | #PO: GIEE0438146 |
| pEZ-M98 | GeneCopoeia | #PO: EX-F0979-M98 |
| pEZ-M98-STAT5 | GeneCopoeia | #PO: EX-F0979-M98 |
| **Software and algorithms** | | |
| IGV | IGV Software | https://data.broadinstitute.org/igv/projects/downloads/2.16/ |
| GraphPad Prism 10.0 | GraphPad Software | https://www.graphpad.com/ |
| Fiji -ImageJ | NIH, USA | https://fiji.sc/ |
